# The Role of Chromosomal Fusions in Genetic Divergence of a Keystone Arctic Species (*Boreogadus saida*)

**DOI:** 10.1002/ece3.74555

**Published:** 2026-10-28

**Authors:** Matthew C. Hale, Natasha Howe, Tracey Loewen, Kevin Hedges, Maxime Geoffroy, Jennifer L. Herbig, Wes Larson

**Affiliations:** ^1^ Department of Biology Texas Christian University Fort Worth Texas USA; ^2^ Pacific States Marine Fisheries Commission Portland Oregon USA; ^3^ National Oceanic and Atmospheric Administration National Marine Fisheries Service, Alaska Fisheries Science Center, Auke Bay Laboratories Juneau Alaska USA; ^4^ Arctic Fisheries and Marine Mammal Science Division Fisheries and Oceans Canada Winnipeg Manitoba Canada; ^5^ Center for Fisheries and Ecosystems Research Fisheries and Marine Institute of Memorial University of Newfoundland St. John's Newfoundland Canada

**Keywords:** chromosome fusion, chromosome inversion, gene flow, genetic divergence, selection

## Abstract

Local adaptation in species with high gene flow can be facilitated through different genomic mechanisms. For example, multiple studies have demonstrated that chromosomal inversions (CIs) are important for adaptive divergence in systems with high gene flow because they help protect locally adaptive alleles from recombination. Although much focus has been given to the importance of CIs in harboring adaptive alleles, there are other mechanisms that can facilitate genetic divergence. Chromosomal fusions can also promote local adaptation in the face of high gene flow by bringing together previously unlinked loci. Herein, we utilized a newly available reference genome for Arctic cod ( *Boreogadus saida* ), together with whole‐genome sequence data from locations spanning a large portion of the Canadian Arctic, to describe the genomic architecture in this circumpolar fish. The Arctic cod genome contains many CIs and five chromosomes that appear to have undergone lineage‐specific chromosomal fusions. Consistent with previous studies, we documented both high gene flow and signals of non‐neutral divergence at a relatively small subset of loci that are driving local population structure. However, our analyses illustrate that most non‐neutral loci are found on fused chromosomes, suggesting chromosomal fusions might be important for local adaptation in this species. This result is especially notable given the localization of divergence in the fused chromosomes when the Arctic cod genome has numerous CIs that are infrequently utilized for divergence across the locations sampled in this study. Our study strongly suggests that non‐neutral loci are concentrated on chromosomal fusions, which may facilitate localized adaptation despite high levels of gene flow in a non‐model species.

## Graphical abstract

Although much focus has been given to the importance of chromosome inversions in harboring adaptive alleles in non‐model species, there are other mechanisms that can facilitate divergence. Here we utilize whole genome sequence data from Arctic cod ( *Boreogadus saida* ) to investigate patterns of adaptive divergence in a system with high gene flow. We find that outlier regions are clustered in chromosomes that underwent multiple fusion events, suggesting that fused chromosomes can also harbor adaptive alleles.

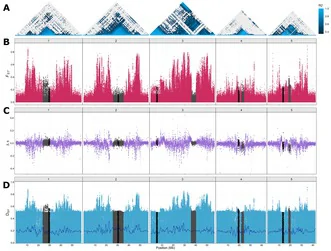

## 1 Introduction

Understanding patterns of genomic differentiation can provide insights into the mechanisms that have allowed organisms to adapt to their environments and how they may evolve in response to future environmental change (Nosil et al. 2009; Stapley et al. 2010; Hohenlohe et al. 2020). Previous empirical and simulation studies have indicated that variation in recombination rate plays a major role in shaping the landscape of adaptive variation (Renaut et al. 2013; Ortiz‐Barrientos et al. 2016; Martin et al. 2019). This is especially true in species with high gene flow, where recombination can disrupt co‐adapted alleles and therefore suppression of recombination can lead to the clustering of adaptive loci (Tigano and Friesen 2016; Berner and Roesti 2017). For example, Samuk et al. (2017) analyzed genomes from 1300 stickleback ( *Gasterosteus aculeatus* ) and found that loci associated with adaptive differentiation are more likely to be found in areas of low recombination when adaptive divergence co‐occurred with high gene flow.

Patterns of recombination within chromosomes are highly variable. Typically, recombination is lower near centromeres and elevated near telomeres (Nachman 2002; McVean et al. 2004). Recombination can also be influenced by structural rearrangements such as chromosomal inversions (CIs), chromosomal fusions, and chromosomal fissions (Yeaman 2013; Näsvall et al. 2023). Recombination between inverted haplotypes in CIs is rare due to the non‐viability of recombinant gametes, and multiple studies have documented clustering of adaptive loci within CIs due to their ability to “protect” adaptive loci by reducing crossover events (Schaal et al. 2022; Wellenreuther and Bernatchez 2018). In contrast, chromosomal fusions are hypothesized to facilitate adaptive divergence by bringing together previously unlinked loci (Guerrero and Kirkpatrick 2014; Wellband et al. 2019). Chromosomal fusions also alter the nuclear structure during meiosis and lead to reductions in recombination per base pair compared to unfused chromosomes (Vara et al. 2021). Moreover, chromosomal fusions have been shown to affect the location of chiasmata suggesting fusion events have a direct effect on the recombination landscape (Vara et al. 2021). A recent study on stickleback provided strong evidence that loci associated with adaptive divergence, including the well‐characterized *Eda* locus, are clustered on chromosomes that have undergone recent fusions (Liu et al. 2021). Although there is now a plethora of literature that documents the importance of CIs in reducing recombination and facilitating adaptive divergence, far fewer studies provide clear evidence of the roles of chromosomal fusions (but see Wellband et al. 2019; Liu et al. 2021; Mackintosh et al. 2023).

Much concern has recently been generated regarding the negative effects of climate change and subsequent reductions in sea ice on Arctic cod ( *Boreogadus saida* ), an abundant circumpolar pelagic fish that is a keystone species in Arctic Ocean food webs (Welch et al. 1992; David et al. 2016; Drost et al. 2016; Laurel et al. 2018). For example, recent data from the Chukchi Sea suggest the range of Arctic cod has shifted north, presumably due to warmer water temperatures, a reduction in winter sea ice, and possible competition from other gadids (i.e., Pacific cod; *Gadus macrocephalus* ) and walleye pollock ( *G. chalcogrammus* ; Wildes et al. 2022). Rapid environmental changes in the Arctic could influence longstanding patterns of connectivity, population structure, and adaptive diversity in Arctic cod. Population genetics studies on Arctic cod can play a vital role for understanding existing population structure and to investigate adaptive divergence that may make certain populations more resilient to future conditions.

There has been a recent flurry of genomics research focusing on both population structure and developing genomic resources in Arctic cod (Bringloe et al. 2024; Maes et al. 2025; Hoff et al. 2026). For example, Bringloe et al. (2024) genotyped 11,233 SNPs in 511 individuals sampled across the Canadian Arctic and identified three genetic groups: (i) the Western Canadian Arctic, (ii) the Eastern Canadian Arctic, and (iii) Hudson Bay area (Hudson Bay, Newfoundland‐Labrador, and the Gulf of St. Lawrence). Maes et al. (2025) genotyped 922 SNPs in 611 individuals across the Arctic Ocean from the Chukchi Sea to Iceland and documented three genetic groups: (i) Beaufort‐Chukchi Seas, (ii) all areas connected by transpolar drift from the Laptev Sea to Iceland, and (iii) West Greenland. These datasets suggest that more southern populations, especially those found in the eastern Canadian Arctic and in Western Greenland, are genetically distinct from northern populations connected by transpolar drift. Both Bringloe et al. (2024) and Maes et al. (2025) identified and conducted analyses separately for both neutral loci and highly differentiated loci that were putatively under selection. In both studies, signals of genetic structure were largely concentrated in candidate adaptive loci with minimal signals of structure remaining in the neutral datasets. However, the investigation of the genomic landscape of adaptive divergence was limited because of the low number of markers genotyped and the absence of a reference genome (Bringloe et al. 2024; Maes et al. 2025).

An additional study constructed a reference genome for Arctic cod and further compared the genome to other gadids (Hoff et al. 2026). This genome characterization provides a vital resource for investigating non‐neutral loci that might be subjected to adaptive divergence in this species. Hoff et al. (2026) found that the Arctic cod genome contains numerous intraspecific CI. Additionally, Hoff et al. (2026) found that Arctic cod contain five species‐specific chromosomal fusions that reduced the chromosome number from 23 (the presumed ancestral number in gadids) to 18. The authors hypothesized that these fusions may have played a role in adaptation to high latitude conditions when Arctic cod diverged from other gadids and colonized new environments.

To that end, the primary goal of our study was to investigate the genomic landscape in Arctic cod across a large portion of their distribution from the Chukchi Sea to Iceland. More specifically, we utilized whole genome sequence data to (a) determine patterns of gene flow, (b) identify non‐neutral—and therefore putatively adaptive—loci, and (c) determine if non‐neutral loci are clustered in regions of the genome associated with chromosomal rearrangements such as fusions or CIs. We observed that most regions of divergence were found on fused chromosomes, with only a few other regions of divergence present on unfused chromosomes that were localized to two CIs. Our study highlights the evolutionary importance of chromosomal fusions and provides compelling evidence for the role of fusions in non‐neutral divergence.

## 2 Materials and Methods

### 2.1 Sample Collection, DNA Extraction, and Genome Sequencing

A total of 172 samples were available for this project from the following three sources: (1) tissue samples from four research vessel programs (all samples from Canadian waters), (2) tissue samples from National Oceanic and Atmospheric Administration bottom trawl surveys conducted in 2012 (Chukchi Sea samples), and (3) previously published whole genome sequences available from Hoff et al. (2024) (samples from Iceland; Figure 1). Canadian samples from Pond Inlet, Broughton Island, and Frobisher Bay were collected using a Campelen 1200 bottom trawl on the M.V. Nuliajuk; the trawl was fished for 15 min on bottom at a speed of 2–3 knots. Sampling near Southampton Island was conducted as part of the Southampton Island Marine Ecosystem Project; a 3 m benthic beam trawl was towed at 2–3 knots for 15 min on bottom at each station. In the Coronation Gulf, Arctic cod were collected as part of a community marine fish monitoring program as part of the Nunavut Shrimp Research Foundation. Sampling from the Labrador Sea and Hudson Strait areas were collected aboard the CCGS *Amundsen* with an Isaacs‐Kidd Midwater Trawl (IKMT) and a beam trawl. The IKMT had 4.5 m × 3.5 m opening and 12.7 mm mesh cod‐end and the beam trawl had a 4.3 m footrope and a net with a 6.3 mm cod‐end mesh. Both nets were towed at three knots. Arctic cod were collected with benthic or beam trawl sampling techniques at various depths (200–750 m) and frozen for processing (see Table 1 for details on sampling locations). Sampling was done irrespective of age (i.e., mature versus immature) and sex.

**FIGURE 1:**
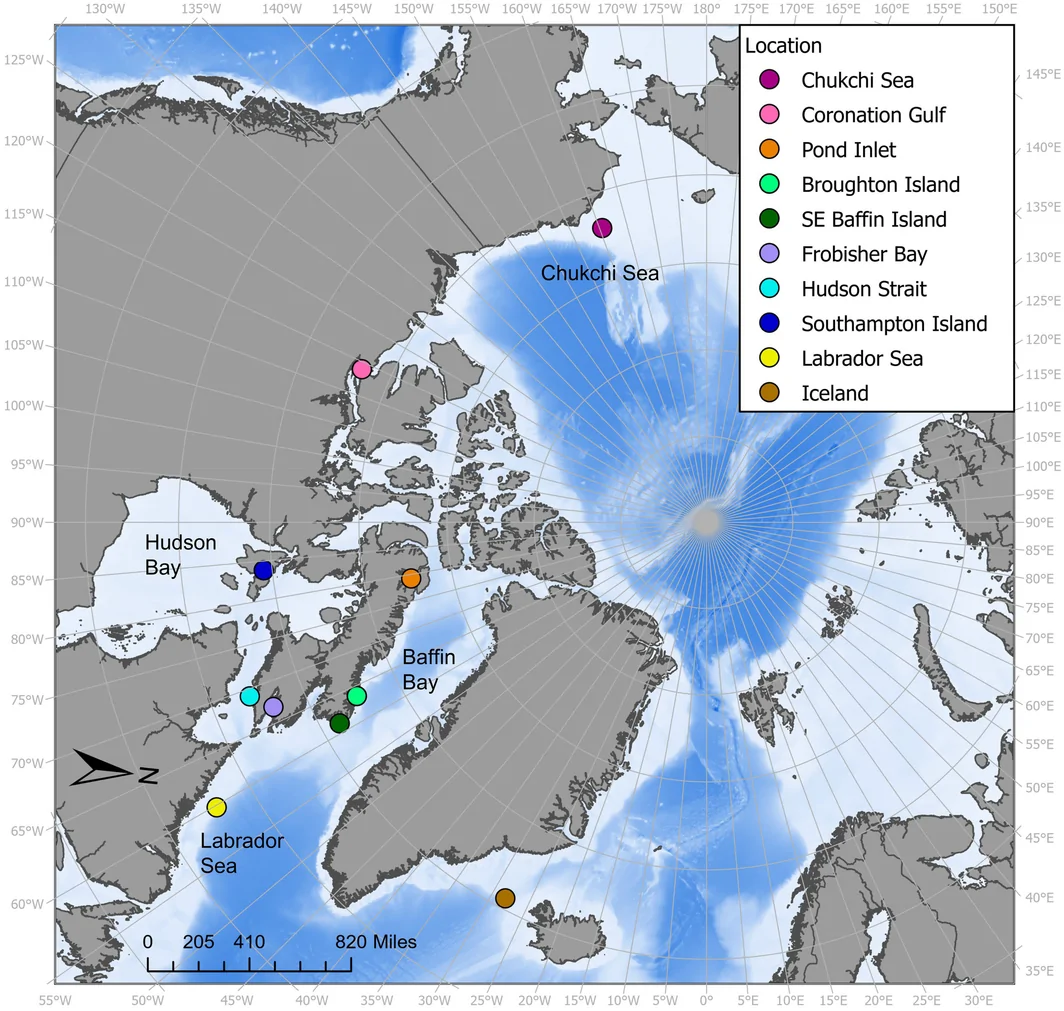
Circumpolar map of *Boreogadus saida* collections included in this study. Samples from Iceland were sequenced in Hoff et al. (2024).

**TABLE 1:** Sampling locations and sample size details of Arctic cod used in this study. Geographic coordinates given are an estimate as many samples were taken from pelagic trawling. Sample sizes given were post sequence filtering and so do not include suspected polar cod ( *Arctogadus glacialis* ).

Collection	Sample size	Latitude	Longitude
Chukchi Sea	36	72.0015	−160.47
Coronation Gulf	12	68.3392	−113.9501
Pond Inlet	26	72.6601	−79.2377
Broughton Island	15	67.5905	−63.587
SE Baffin Island	13	66.0347	−61.2705
Frobisher Bay	18	63.0714	−71.3192
Hudson Strait	14	62.142	−69.14268
Southampton Island	13	64.5319	−83.8164
Labrador Sea	7	57.9013	−59.8065
Iceland	18	65.5793	−28.1558

For newly collected samples, DNA was extracted from fin clips stored in ethanol with a standard phenol‐chloroform extraction (Gautam 2022). DNA was quantified using a Nanodrop (Thermo Fisher Scientific, Waltham, MA, USA) and standardized to 50 ng/μl of DNA. DNA quality was also checked on a 2% agarose gel. Samples with high‐quality DNA bands were selected for whole‐genome sequencing. Briefly, 1 mg of DNA was submitted to Novogene (Sacramento, CA, USA) and prepared for sequencing using Illumina (San Diego, CA, USA) TrueSeq library kits. Libraries were sequenced using paired‐end 150 bp sequencing on an Illumina NovoSeq S4 sequencer. The genome size of Arctic cod is ~585 MB (Hoff et al. 2024), and we sequenced the individuals on four lanes to achieve a target coverage of ~10× per individual.

### 2.2 Sequence Alignment and Genotype Likelihood Estimation

FASTQ reads were trimmed to remove Illumina adaptors and poor‐quality sequenced bases using Trimmomatic V 0.32 (Bolger et al. 2014). Trimmed reads were then aligned to a draft version of the Arctic cod genome (GCA_964260565.1; Hoff et al. 2024) using BWA‐MEM v0.7.17 with default parameters (Li and Durbin 2009; Li 2011). The aligned reads were then processed with SAMtools v1.18 (Li 2011) and converted to sorted bam files using default parameters. Mean depth of coverage was also calculated for each sample [samtools depth]. Subsequently, the Iceland samples were downsampled from their original ~20× depth to match the average ~10× depth of the North American Arctic samples sequenced in this study. Genotype likelihoods (GLs) were calculated for US/Canadian Arctic populations in ANGSD v0.930 (Korneliussen et al. 2014) with the SAMtools model [GL 1]. The minimum minor allele frequency was set to 1% [minMaf 0.01], and a *p*‐value cutoff of 10^−10^ was used to remove rare alleles and low confidence SNPs [snp_pval 1e−10]. The minimum number of individuals with genotype likelihoods at a polymorphic locus was set to 2/3 the sample size [minInd 102], minimum depth of coverage was set to five times the sample size [setminDepth 770], and maximum depth was set to the sample size multiplied by twice the depth of coverage (~10×), to account for individuals and loci with greater coverage [setmaxDepth 3080]. Genotype likelihoods with at least a 99.9% base call accuracy [minQ 30] and mapping accuracy [minMapQ 30] were retained. To counteract batch effects, downsampled Iceland sequences were incorporated into a new genotype likelihood run with the same filters as above after adjustment for the increased sample size and the addition of the ‐sites flag to limit GLs to the previously identified SNPs in the US/Canadian Arctic populations (Lou and Therkildsen 2022).

### 2.3 Genome‐Wide Population Genetic Analysis

To explore genetic divergence across collections, principal component analyses (PCAs) were conducted in PCAngsd v1.34.6 (Meisner and Albrechtsen 2018). Five outlier samples in initial whole genome PCAs suggested the presence of five polar cod ( *Arctogadus glacialis* ), which were removed from all subsequent analyses (c.f. Bringloe et al. 2024). This ratio of polar cod to Arctic cod in Canadian waters is similar to that found by Bouchard et al. (2016). After removing suspected polar cod individuals, PCAs were reanalyzed for the whole genome and each chromosome, as well as in the sex‐associated region (OZ177908.1:26000000–27500000) identified in Hoff et al. (2024), to examine patterns of population structure and ensure that our results were not biased by unequal sex ratios. Additionally, whole genome admixture was evaluated with NgsAdmix (Skotte et al. 2013) for two to six clusters (*K*), and optimum *K* values were inferred from maximum likelihood scores across 10 runs per cluster. We then calculated weighted pairwise *F*_ST_ across all loci for collections with greater than 10 individuals to avoid imprecise site allele frequency estimates (Lou et al. 2021), and therefore Labrador Sea was not included (*n* = 7). *F*_ST_s were calculated by first creating site allele frequency likelihoods for samples from each collection in ANGSD using the same filtering criteria used to call GLs except for the removal of snp_pval and minMaf because SNPs were restricted to those from the original all‐collection GL run [‐sites].

Manhattan plots of pairwise *F*_ST_s for each locus were used to visualize genome‐wide differentiation between collection comparisons. A striking pattern of elevated pairwise *F*_ST_s in chromosomes 1 through 5 was apparent, especially in *F*_ST_ comparisons including samples from the Chukchi Sea. We also performed a selection scan using PCAngsd's FastPCA [‐‐selection –site‐save ‐‐weights], using a False Discovery Rate cutoff (alpha = 0.001), and almost all outlier loci on PC1 were attributed to the first five chromosomes (99.4%). These five chromosomes likely underwent fusions during or after speciation from an ancestral gadid (Hoff et al. 2026). Due to the evident dichotomy of differentiation between the fused chromosomes (chrs1‐5) and unfused chromosomes (chrs6‐18), we split analyses by these two chromosome types to further investigate patterns of gene flow and adaptive divergence. Additionally, we explored differences in population structure between neutral and non‐neutral loci to emulate the results of two previous studies (Maes et al. 2025; Bringloe et al. 2024) that identified variable patterns of population structure between these types of markers in Arctic cod.

### 2.4 Investigation of Patterns of Genetic Differentiation in Fused and Unfused Chromosomes and Between Neutral and Non‐Neutral Loci

We created a series of datasets to examine patterns of genetic differentiation within and outside fused regions at putatively neutral and non‐neutral loci. These datasets were: (1) fused chromosomes with all loci, (2) fused chromosomes with non‐neutral loci, (3) unfused chromosomes with all loci, (4) unfused chromosomes with neutral loci, and (5) unfused chromosomes with non‐neutral loci. We did not attempt to identify neutral loci in the fused chromosomes because the breadth of high *F*_ST_ across these chromosomes made it difficult to confidently identify loci that would not be linked to other highly differentiating loci. We calculated average weighted *F*_ST_ for each location pair separately for the fused and unfused chromosomes (datasets 1 and 3 above) in ANGSD.

Second, we identified neutral and non‐neutral marker sets from the unfused chromosomes and a non‐neutral marker set from the fused chromosomes. Pairwise *F*_ST_ values for each locus were transformed into *Z*‐scores to derive a metric that could be used to statistically identify neutral and non‐neutral loci. *Z*‐score transformations were conducted separately for the unfused and fused datasets because the distribution of *F*_ST_s for these datasets was different. Bonferroni‐corrected *p*‐values (𝜶 = 0.05, correction based on number of loci) were converted to *Z*‐scores using the R package *NCmisc* (Cooper 2022; R Core Team 2023). The unfused chromosomes were separately run in PCAngsd's FastPCA [‐‐sites‐save ‐‐weights ‐‐selection], and a False Discovery Rate (𝜶 = 0.001) was applied to the *p*‐values to determine a neutral marker cutoff (Meisner et al. 2021). To best visualize the neutral structure, loci in putative CIs identified in Hoff et al. (2024, 2026) and in this study were removed. From these filtered markers, neutral loci in the unfused chromosomes were identified as those both below the FastPCA selection threshold and with *Z*‐scores consistently below the significance thresholds in every location comparison. Loci with a significant *Z*‐score (scores greater than the *Z*‐score corresponding to the Bonferroni‐corrected *p*‐value) in at least one location comparison were considered non‐neutral and were separated by fused and unfused chromosomes. PCA and admixture analyses were conducted on each of the five datasets described above to visualize patterns of population structure.

### 2.5 Characterizing Recombination Landscape, Genomic Architecture, and Diversity

Linkage disequilibrium (LD) was calculated to investigate patterns of recombination across the genome. Genotype calls were determined in ANGSD [doPost 1 doGeno 2 doPlink 2], and genotypes were removed if the depth was less than five for each individual [geno_minDepth 5]. LD was calculated across the genome with PLINK v1.9 (Chang et al. 2015; Purcell et al. 2007) using the following command [‐‐ld‐window‐r2 0 ‐‐r2 inter‐‐chr]. Subsequent pairwise estimates of *r* ^2^ were filtered for *r* ^2^ > 0.35 (to reduce computational load and aid in locating regions of the genome with elevated LD) and plotted separately for each chromosome in R 4.3.1 (2023) using the ggplot2 package (Wickham 2016).

We used the R package *Lostruct* (Li and Ralph 2018) to locate regions of the genome with patterns of population structure that deviate from the genomic background due to factors such as selection and/or structural variation (see Huang et al. 2020). Genotype data were first converted to a BCF file using R‐code and BCFtools v1.18 (Li 2011). The genome was divided into nonoverlapping windows of 100 SNPs, and local PCAs were created for each window. These windows were then compared to each other using Euclidean distances, and differences between samples were then mapped into 40 multidimensional scales (MDS). We determined MDS outliers with a *Z*‐test statistic, using two standard deviations around the mean (𝜶 = 0.001) and required at least 10 consecutive outlier windows.

Although MDS outliers can indicate the presence of CIs, they can also be the result of hitchhiking selection in regions of the genome with low levels of recombination (Li and Ralph 2018). Therefore, we classified outlier regions as putative CIs if they (a) produced 10 or more consecutive MDS outliers along the same axis, (b) exhibited elevated linkage disequilibrium (i.e., linkage block with *r* ^2^ values > 0.35), (c) produced three discrete clusters in a local PCA, and (d) showed elevated heterozygosity within the candidate region in individuals presumed to be heterozygous for the CI karyotype. Local PCAs within a CI are expected to have three discrete clusters corresponding to the three CI karyotypes due to extensive recombination suppression between homozygous karyotypes. More complex inversions, like double inversions, may present as six or nine clusters. The latter were not considered putative inversions due to the difficultly to diagnose but were noted as potential complex inversions in Table 2. To that end, we used PCAngsd to calculate PCAs for loci within all candidate MDS outlier regions to test if they formed three discrete clusters. We also tested the assumption that the intermediate karyotype (i.e., cluster) should display elevated heterozygosity compared to the two putative homozygotes (Huang et al. 2020). Therefore, we used PLINK v1.90b7 to calculate heterozygosity for loci within the MDS outlier region and compared individuals from the central PCA cluster (the putative heterokaryotype) to individuals from the exterior PCA clusters (putative homokaryotypes) using a Wilcoxon test and a Benjamini‐Hochberg *p*‐value adjustment (𝜶 = 0.05), expecting that the homozygous groups would have lower heterozygosity than the central group. Lastly, MDS outlier regions were compared to LD heatmaps to determine if they were located within a high linkage block. If the MDS outlier followed the expectations of a CI for all three tests, they were considered putative CIs. We also compared the size and location of putative CIs with Hoff et al. (2026). The locations of candidate CIs were further compared to regions of elevated genetic differentiation (i.e., *F*_ST_ values) to determine if CIs could be involved in local adaptation.

**TABLE 2:** MDS outliers were evaluated to determine whether they met putative CI criteria. All regions listed in the table contained at least 10 consecutive MDS outlier windows, and those that passed all putative CI tests were considered putative CIs. Putative CI boundaries are assigned based on MDS outliers and therefore do not always align to that of Hoff et al. (2026) nor to all the CIs identified in that study presented here.

Chr	Position (Mb)	*F*_ST_ outlier	Number of clusters on PC1	Elevated heterozygosity in middle PCA cluster	Elevated LD	Pass all CI tests	Inversion name in Hoff et al. (2026)
1	21.92–23.01	No	Unclear	Yes	Yes	No	
1	25.1–30.7	No	Unclear	No	Yes	No	
1	31.1–34.28	No	3	No	Yes	No	
2	24.5–37.1	No	Unclear	Yes	Yes	No	Bschr2
2	46.2–48.9	Yes	3	Yes	Yes	Yes	
3	27.8–30.8	Yes	3	Yes	Yes	Yes	
3	34.2–35.9	Yes	3	Yes	Yes	Yes	Bschr3
4	20.4–25.5	No	Unclear	No	Yes	No	
5	18.3–18.9	No	3	Yes	Yes	Yes	Bschr5
5	25.7–27.6	No	2	No	Yes	No	Sex region
6	19.1–23.1	No	3	Yes	Yes	Yes	Bschr6.01
7	10.7–19.4	No	Unclear	Yes	Yes	No	Bschr7.02
8	17.3–20.8	No	Unclear	Yes	Yes	No	
9	22.04–23.9	No	3	Yes	Yes	Yes	Bschr9
10	0.4–8.5	No	3	Yes	Yes	Yes	Bschr10
11	0.1–8.7	No	Unclear	No	Yes	No	
12	0.05–2.9	No	3	Yes	Yes	Yes	Bschr12
13	0.02–3.9	No	3	Yes	Yes	Yes	Bschr13 (partA)
13	9.04–15.4	No	3	Yes	Yes	Yes	Bschr13 (partB)
14	5.1–10.4	No	3	Yes	Yes	Yes	Bschr14
15	9.06–14.3	No	3	Yes	Yes	Yes	Bschr15
16	6.2–8.5	No	Unclear	Yes	Yes	No	Bschr16.02
17	6.79–17.2	No	3	Yes	Yes	Yes	Bschr17

Genetic diversity was measured within and between Chukchi Sea and Pond Inlet to better understand possible adaptive signals within fused chromosomes. These populations were selected for three reasons: (1) they had the greatest sample sizes, (2) Chukchi Sea is the most geographically isolated population, and (3) Chukchi Sea and Pond Inlet have elevated genetic differentiation based on *F*_ST_ results (see Figure 2). Nucleotide diversity was calculated in ANGSD [realSFS saf2theta thetaStat do_stat] for Chukchi Sea and Pond Inlet with a window size of 20,000 base pairs and a step size of 10,000 base pairs. We then calculated delta *pi* (Δ𝝅 = 𝝅_Pond_ − 𝝅_Chukchi_) to determine whether signatures of adaptation were similar in both populations across the fused chromosomes (following analyses for selection in fused chromosomes conducted in Liu et al. 2021). Absolute genetic divergence (*D*_XY_) was calculated for each SNP using the calcDxy.R script available in ngsPopGen (Fumagalli et al. 2014) and additionally visualized with a sliding window of 500 kb windows and 100 kb steps, to further explore the mechanisms behind the observed genetic differentiation. Centromere locations and fusion points were estimated based on Hoff et al. (2026), from the Hi‐C maps and synteny plots (see corresponding Figure S1 and Figure 2, respectively) and were plotted alongside diversity metrics. Since the identified centromere locations were denoted in Hi‐C maps with a single arrow for each chromosome, all centromeres were estimated to be 1 Mb wide for consistency.

**FIGURE 2:**
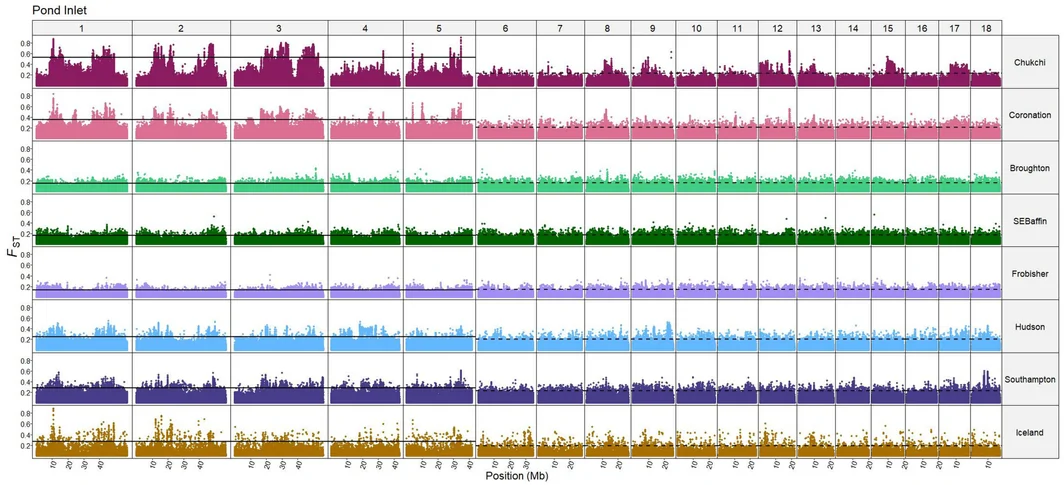
Manhattan plots of whole genome genetic differentiation (*F*_ST_) of comparisons between collections sampled in this study compared to Pond Inlet (except Labrador Sea because of low sample size). Horizontal lines represent the Bonferroni‐corrected 𝘱‐value (α = 0.05) from the *Z*‐score distribution of *F*_ST_ separately calculated for the fused (solid) and unfused (dashed) chromosomes that were used to determine the non‐neutral and neutral loci.

## 3 Results

### 3.1 Sequencing Metrics and Initial Investigation of Population Structure and Genetic Differentiation

After removing two individuals with less than 1× coverage, whole genome sequencing of 154 samples of North American Arctic cod had an average depth of 9.31× (range = 5.62×–14.21×: Table S1). Together with 18 samples from Iceland (Hoff et al. 2024), a total of 1,792,130 SNPs remained after filtering for minor allele frequencies less than 5% and 33% missing data.

Exploratory population genetic analyses (PCA, admixture, and pairwise *F*_ST_) with the full dataset revealed that the largest genetic divergence occurred between the Chukchi Sea samples and all collections from Canadian waters besides the Coronation Gulf (Figures 2 and 3 and Figure S1, Appendix A). Interestingly, the Iceland population clustered between the Chukchi Sea and the Canadian Arctic populations. Although sample sizes are small from the Labrador Sea, PCA and admixture analyses suggest that this collection, along with many of the individuals near Hudson Bay in the southern Canadian Arctic, were genetically differentiated from other Canadian Arctic collections (Figure S1, Appendix B). The best fit model based on maximum likelihood scores for the whole genome admixture analysis was *K* = 2, reflecting the high differentiation between the populations from the Chukchi Sea and other populations (Appendix B, whole genome admixture). However, results from runs with higher *K*‐values (i.e., *K* = 4) displayed similar visual patterns of structure as the PCA, just with more separation (e.g., some of the Hudson Bay samples).

**FIGURE 3:**
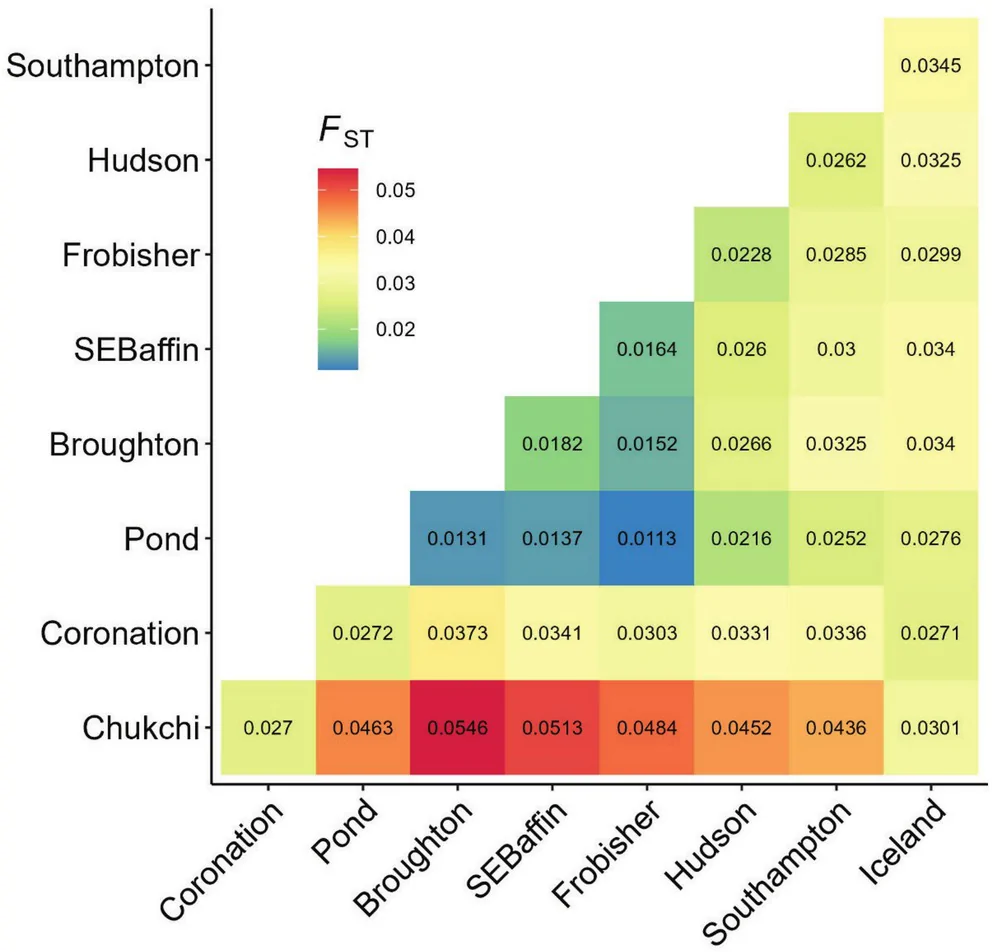
Heatmap of weighted *F*_ST_ across the whole genome between collections. Labrador was not included due to low sample size.

PCAs constructed for each chromosome revealed strong evidence for structural variants, many of which were also reported in Hoff et al. (2024, 2026), and many chromosomes displayed discrete clusters not associated with population structure (Figure S2; discussed in more detail below). A local PCA of the sex‐associated region parsed individuals across both PC1 and PC2, and sex ratios were estimated by the PC1 split (Figure S3). Hoff et al. (2024) found two male haplotypes that likely explain the similar pattern of variation on PC2, especially considering the increase in heterozygosity in the putatively male individuals of both haplotypes. Southampton Island, Chukchi Sea, and Hudson Strait had more individuals in the negative PC1 space (64%, 71%, and 76%, respectively) that likely correspond to males while the remaining populations had an approximately equivalent sex ratio. The sex‐associated region did not display a clear pattern of elevated *F*_ST_ values, indicating that variable sex ratios did not produce false signals of adaptive divergence (Figure 2).

Whole genome *F*_ST_ estimates suggest high levels of gene flow between many populations in the Canadian Arctic, especially populations near Baffin Bay that not only have low average *F*_ST_ but also few regions of elevated *F*_ST_ across the genome (Figures 2 and 3 and Appendix A). The lack of *F*_ST_ peaks in these populations contrasts with the numerous genomic regions that are elevated when comparing Pond Inlet to the two westernmost populations, that is, Chukchi Sea and Coronation Gulf. Interestingly, Iceland shares some but not all these regions of differentiation with the Chukchi Sea, possibly explaining Iceland's more centralized clustering in the whole genome PCA. The majority of these elevated *F*_ST_ regions correspond to the five fused chromosomes, with *F*_ST_ values exceeding 0.8 in numerous population comparisons (max *F*_ST_ = 0.92). Although there are still elevated estimates of *F*_ST_ in some genomic regions on the unfused chromosomes, differentiation is more sporadic, localized, and lower overall. Similarly, the selection scan identified outlier markers almost exclusively in the fused chromosomes for PC1 (99.4% of outlier SNPs for PC1 and 55.9% for PC2; Figure S4).

### 3.2 Patterns of Genetic Differentiation at Fused and Unfused Chromosomes and Between Putatively Neutral and Non‐Neutral Loci

Weighted *F*_ST_ values for population compared to the Chukchi Sea were ~2.5 times greater for markers in fused chromosomes compared to unfused chromosomes (average weighted *F*_ST_ for Chukchi comparisons = 0.027 for unfused chromosomes and 0.068 for fused chromosomes, Figure S5). Contrastingly, weighted *F*_ST_ values for most populations in the Canadian Arctic were similar between fused and unfused chromosomes. Furthermore, patterns of population structure from markers at fused and unfused chromosomes differed substantially (Figure 4 and Figure S5). While Chukchi Sea is diverged from other populations in both fused and unfused datasets, the main signal of variation in unfused chromosomes is likely due to putative CIs. Specifically, the PCA constructed from markers on unfused chromosomes displays three discrete clusters that include individuals from across the sampling range, with slight separation of Chukchi Sea from other populations at the edge of each cluster (Figure 4A). Contrastingly, the fused marker dataset reflects the population structure from the whole genome dataset (see Figure S1) with clustering generally corresponding to Chukchi Sea, Coronation Gulf/Iceland, Hudson Bay/Labrador Sea, and the rest of the Canadian Arctic (Figure 4D). After removal of putative CIs from the neutral dataset, the patterns of structure were somewhat different than that of the non‐neutral dataset (Figure 4B,E). The variance was expectedly lower in the neutral than the non‐neutral datasets, and Iceland clustered closer with Chukchi in the neutral compared to non‐neutral dataset. Further, the Hudson Bay complex separated out as its own cluster in the non‐neutral admixture more so than in the neutral admixture analysis. For example, analysis of the unfused neutral dataset at *K* = 4 displayed evidence of multiple clusters associated with putative CIs (Figure 4C) whereas the fused non‐neutral dataset at *K* = 4 reflected the genetic groups outlined above (Figure 4F, see Appendix B for admixture at *K* = 5 and *K* = 6). Patterns of population structure in the unfused non‐neutral dataset were similar to those from the fused non‐neutral dataset, except the non‐neutral variation within Iceland did not form a discrete cluster (maximum likelihood of *K* = 3 compared to *K* = 4 in the fused datasets: Figure S6, Appendix B). In summary, genetic variation in the neutral dataset indicates reduced gene flow between Chukchi Sea and Iceland from the other locations, whereas variation in the non‐neutral dataset is associated with finer‐scale spatial structure.

**FIGURE 4:**
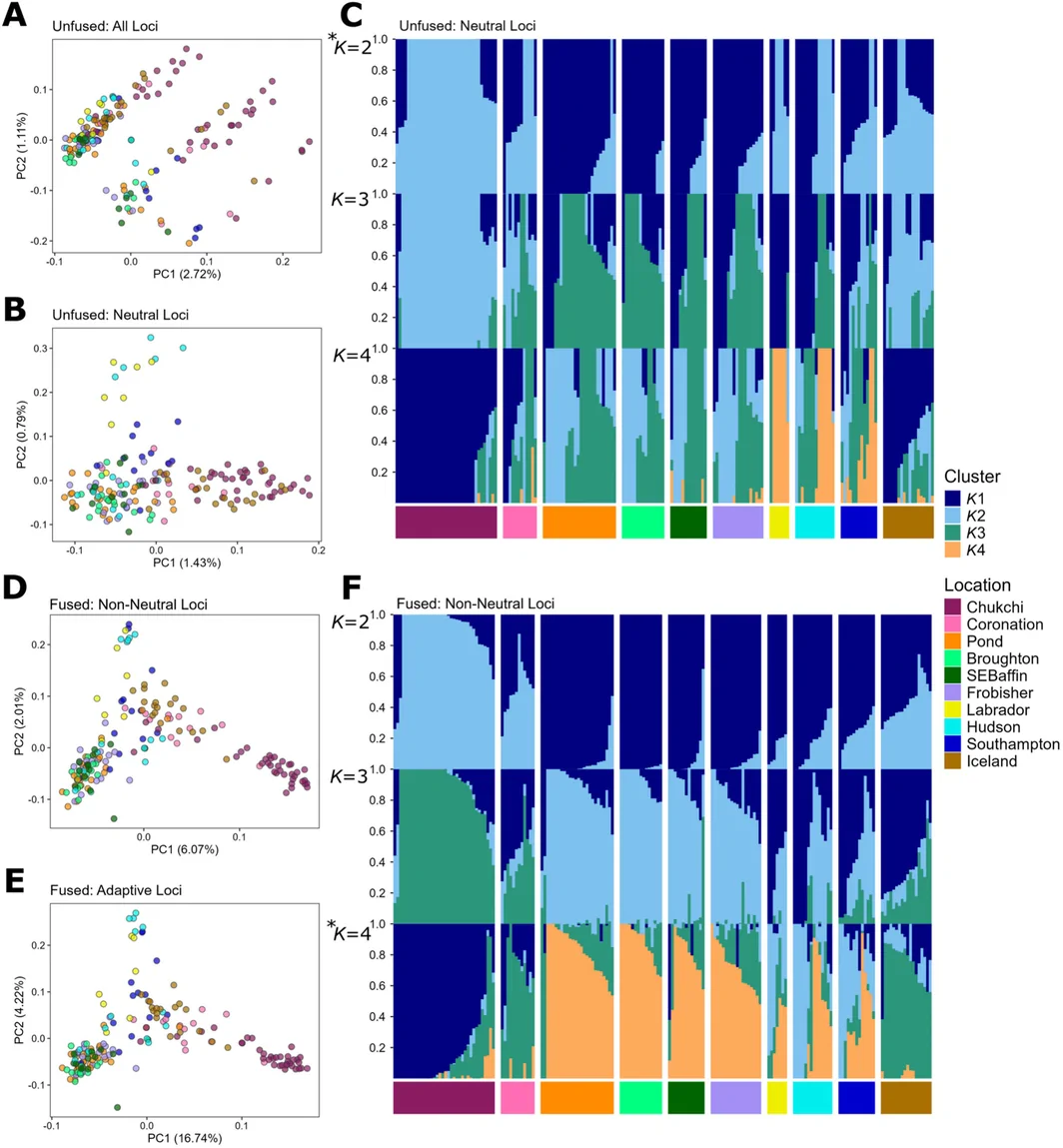
Genetic variation and structure across Arctic cod populations included in this study. Neutral structure was visualized from unfused chromosomes in PCAs for both (A) all unfused loci and (B) only neutral loci (*n* = 812,125) and subsequent (C) admixture of unfused neutral loci for *K*2 to *K*4. Non‐neutral structure was visualized from fused chromosomes in PCAs for both (D) all fused loci and (E) only fused non‐neutral loci (*n* = 55,756) and (F) admixture of fused non‐neutral loci for *K*2 to *K*4. Neutral and non‐neutral loci were determined using a Bonferroni‐corrected 𝘱‐value (α = 0.05) from the *Z*‐score distribution of *F*_ST_ across the unfused and fused chromosomes, respectively. Neutral markers were additionally pruned to remove putative structural variants (see Methods). Asterisks on admixture plots indicate the maximum likelihood *K*‐value.

### 3.3 Investigating Recombination Landscape and Identifying Putative Structural Variants

We identified 23 outlier regions using Lostruct's MDS analyses. At least one outlier region was found on every chromosome except chromosome 18, suggesting a strong presence of structural variants within the Arctic cod genome (Figure 5). These outlier regions were further investigated for signals of putative CIs and compared to the genomic locations of CIs reported in Hoff et al. (2026; Table 2). Of the 23 outlier regions investigated, one was identified as the sex region and 13 were classified as putative CIs after meeting three additional criteria (elevated LD, distinct PCA clusters, and elevated heterozygosity of putative heterozygous karyotype) on chromosomes 2, 3, 5, 6, 9, 10, 12–15, and 17; Table 2; Appendices C and D; and some matched the CIs reported in Hoff et al. (2026). We also found evidence suggestive of potential complex or double CIs at multiple MDS outliers that were filtered out of our analysis. Several outlier windows either created two clusters, where the third genotype is likely not present, or six clusters that could be representative of multiple inversions within the outlier region. For example, Hoff et al. (2026) identified regions on Bschr7.02 and Bschr16.02 as candidate CIs; however, we were hesitant to consider them CIs due to low sample sizes and PCAs from loci within these regions producing six discrete clusters (Table 2, Appendix C). In addition, loci from four more candidate CIs—on chromosomes 1, 2, and 7—produced two rather than three distinct clusters in local PCAs suggesting that there has either been recombination within homokaryotypes or that not all three karyotypes are present in the sampled individuals (Table 2 and Figure 5). There are multiple putative CIs that were identified in the fused chromosomes (one on chr 2, two on chr 3, and one on chr 5) that all differentiated Chukchi Sea samples from most other populations, whereas only two of the remaining 11 putative CIs on unfused chromosomes showed clustering by location. The localization of CIs with location‐specific clustering on the first five chromosomes may suggest that there is interplay between chromosomal fusions and the putative CIs that were brought together with these fusion events.

**FIGURE 5:**
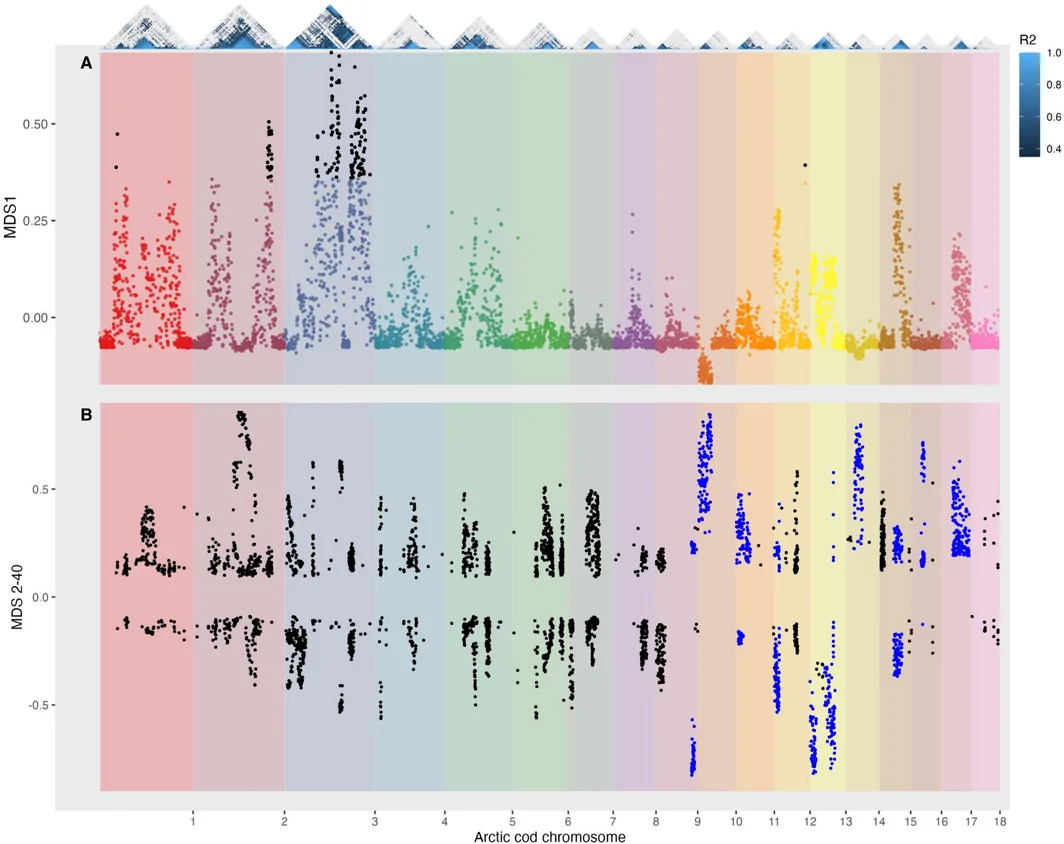
MDS plots for (A) the first dimension with outliers colored in black, where almost all outlier loci are in three of the five fused chromosomes, and (B) collective MDS outliers for dimensions 2–40, with outliers located in putative CIs colored in blue (see Table 2). LD heatmaps for values of *r* ^2^ greater than 0.35 are plotted above each respective chromosome.

The construction of the Arctic cod reference genome and corresponding synteny to gadid species uncovered fusions of the first five chromosomes unique to the evolution of *Boreogadus* (Hoff et al. 2026). Visualizing the landscape of differentiation, recombination, and diversity along with putative locations of centromeres and fusion points on chromosomes 1–5 revealed striking differences between loci within and outside of these regions (Figure 6). Putative centromeric regions and fusion points displayed reduced differentiation in location comparisons and elevated LD compared to other regions on these chromosomes. The pattern of reduced differentiation is especially pronounced on chromosome 3 in the fusion point. There was no observed difference in nucleotide diversity between the populations from Pond Inlet and the Chukchi Sea (Δ*Pi*) at putative centromeres, and differences in fusion points were similarly low (Figure 6). Additionally, we observed reductions in genetic differentiation at the distal regions of each of these three chromosomes that correspond to little difference in nucleotide diversity between Pond Inlet and Chukchi.

**FIGURE 6:**
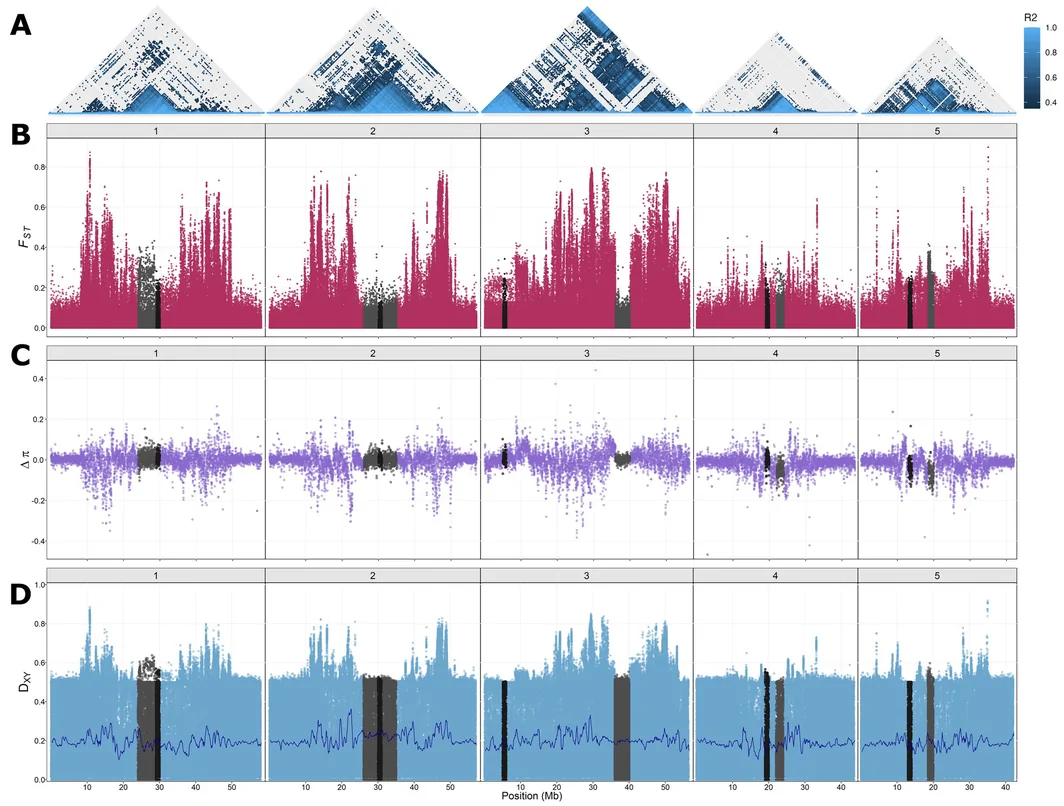
Diversity statistics for fused chromosomes 1–5 showing (A) linkage disequilibrium (*r* ^2^) calculated from all samples except Iceland, (B) genetic differentiation (*F*_ST_) between Pond and Chukchi, (C) the difference in nucleotide diversity (Δ𝝅) between Pond Inlet and Chukchi, where +Δ𝝅 corresponds to greater diversity in Pond and −Δ𝝅 corresponds to greater diversity in Chukchi, and (D) absolute genetic divergence (*D*_XY_) within Pond and Chukchi. A sliding window of 500 kb windows and 100 kb steps was included (blue line) to visualize average *D*_XY_ trends across the chromosomes. Loci predicted to be within putative centromere locations are denoted in black and those predicted to be in the putative fusion regions are denoted in gray (estimated based on Hoff et al. 2026).

Estimates of Δ*Pi* on regions of fused chromosomes that corresponded with elevated genetic differentiation did not show a unidirectional trend of consistently higher nucleotide diversity in one population over the other (Figure 6 and Figure S7). Moreover, elevated *D*_XY_ in regions associated with high *F*_ST_ indicates that differentiation is not due to a reduction in diversity across one population. Rather, the increased diversity in regions of differentiation does not show a unidirectional trend by population, suggesting that differing selective pressures are present in both Chukchi Sea and Pond Inlet that have created a heterogeneous landscape of genomic diversity across these chromosomes.

## 4 Discussion

This study provides evidence for the localization of genetic variation within chromosomal fusions between broad geographic locations for a species that experiences high gene flow. Here we utilized a recently constructed reference genome for Arctic cod (Hoff et al. 2026), along with whole genome sequence data from samples spanning a large portion of the species' range, to investigate population structure and potential mechanisms responsible for genetic divergence. We found that signals of non‐neutral divergence were concentrated in fused chromosomes even with an abundance of putative CIs throughout the Arctic cod genome. Herein, we support findings from two recent studies that found that signals of population differentiation in Arctic cod were largely harbored in non‐neutral loci (Maes et al. 2025; Bringloe et al. 2024). However, here we build on those studies by suggesting that—at least for the populations sampled herein—chromosome fusions are the genomic mechanism promoting adaptive divergence.

### 4.1 Population Structure of Arctic Cod at Neutral and Adaptive Markers

Patterns of population structure inferred from our data were similar to previous studies (Maes et al. 2025; Bringloe et al. 2024), and the partial overlap of our sampling locations is useful for unifying interpretations of structure across geographic regions. In our study, the main discernable population structure at neutral markers was differentiation between collections from Chukchi Sea, Coronation Gulf, and Iceland from the rest of the samples along with subtle evidence of additional differentiation between samples from Hudson Bay, Labrador Sea, and Southampton Island (see Figure 4B). This pattern is confirmed by our admixture analyses, which clusters collections from Chukchi Sea, Iceland, and Labrador Sea separately from the rest of the samples when *K* = 2. Note that our analyses using non‐neutral markers found similar patterns. We hypothesize that genetic structure in this high gene flow species is driven by a combination of factors that influence connectivity (e.g., currents and water circulation) as well as selective pressures from environmental variables such as the presence and distribution of sea ice.

As in many marine species, structure in Arctic cod appears to be at least partially shaped by ocean currents and water circulation patterns in the high Arctic (Maes et al. 2025). For example, Iceland is more genetically similar to the Chukchi Sea and Coronation Gulf samples than to the other more geographically proximate locations in Baffin Bay, Hudson Bay, and Labrador Sea. This is likely because of long distance connectivity facilitated by the Beaufort Gyre, which transports water and ice through the Chukchi Sea and meets the Transpolar Current before flowing towards the Atlantic (Constantin and Johnson 2024; see Maes et al. 2025 for detailed description of currents in relation to Arctic cod movements). Contrastingly, ocean currents in western Baffin Bay, Hudson Bay, and Labrador Sea regions flow south (Constantin and Johnson 2024), and currents are additionally restricted by the land masses of Greenland and Baffin Island. This likely contributes to the genetic structure observed between these eastern Canadian collections and the remaining western Arctic and Iceland collections. Another potential driver of restricted gene flow is the large freshwater input from the Mackenzie River (Mulligan and Perrie 2019), which empties into the Beaufort Sea near the Alaska/Canada border. Restricted gene flow caused by the Mackenzie River could contribute to the genetic divergence observed between the Coronation Gulf and Chukchi Sea collections, but further sampling in and around western Canada and eastern Alaska would help further elucidate genetic structure.

Finer scale variation in current and water circulation patterns in Baffin Bay, Hudson Bay, and the Labrador Sea may also partially explain the somewhat complex patterns of population structure found in collections from this region (Maes et al. 2025). The west Greenland and Baffin Island currents, which flow northwest and southeast respectively (Münchow et al. 2015), likely facilitate gene flow among collections in Baffin Bay (Pond Inlet, Broughton Island, SE Baffin Bay, Frobisher Bay), which are genetically similar to each other, and likely isolate these collections from genetically diverged southern and western populations. No study to date has analyzed collections from both the Canadian and Greenland sides of Baffin Bay, and only one study to our knowledge (Maes et al. 2025) analyzed samples from west Greenland. They found that west Greenland samples were genetically diverged from populations outside of Baffin Bay, and we hypothesize that those populations are genetically similar to samples from the Canadian side of Baffin Bay analyzed herein and by Bringloe et al. (2024) given cyclonic current patterns in the Bay. Restriction of water flow in Hudson Bay and between the Labrador and west Greenland/Baffin Island currents (Straneo and Saucier 2008) likely explains the subtle genetic structure observed among collections in these regions. However, individuals in some of our collections belong to different genetic groups (see Figure 4C). In the case of Hudson Strait, we hypothesize that samples from this collection contained multiple genetic groups because the mouth of the Hudson Bay is a dynamic transition zone where multiple currents meet and are influenced by large tides and other sources of environmental forcing (Straneo and Saucier 2008). Ultimately, the major currents in the Arctic seem to be a strong indicator of the observed neutral genetic structure present in these Arctic cod populations.

The largest genetic divergence in our study was observed at regions of the genome associated with chromosomal fusions. This divergence was particularly pronounced between collections from the Chukchi Sea and those around Baffin Bay. We hypothesize that this concentrated differentiation is possibly the result of selection in response to differing temperatures and sea ice regimes. Baffin Bay is characterized by high variation in temperature and sea ice cover both within and between calendar years and is likely becoming a suboptimal nursery ground for Arctic cod, with recruitment failures documented in warmer years (Bouchard et al. 2020; Maes et al. 2025; Geoffroy et al. 2023). Contrastingly, the Chukchi Sea has historically been largely covered by sea ice in winter, and current patterns suggest that larvae are moved from this region into the Beaufort Gyre, which entrains sea ice, providing Arctic cod with consistent and necessary ice‐associated habitat for their development (Maes et al. 2025; Petty et al. 2016). These environmental differences may coincide with varying genetic adaptations and possibly help explain the genetic differences between these populations.

### 4.2 Landscape of Genetic Differentiation and Importance of Chromosomal Fusions

Areas of high genetic differentiation in our study are concentrated on chromosomes 1–5, all of which underwent lineage specific fusion events in *Boreogadus* (see Hoff et al. 2026; Figure 3 and Table 3). These chromosomes (especially chromosomes 1–3) contain prominent peaks of elevated *F*_ST_ (some regions with *F*_ST_ > 0.8) that span many megabases. Similarly, Bringloe et al. (2024) attributed most of the variation along PC1 in their samples to loci found on chromosomes syntenic to *Boreogadus* chromosomes 1–3 (note, Bringloe aligned genomic data to the Atlantic cod ( *Gadus morhua* ) genome), suggesting that these chromosomes contain loci that are consistently variable between populations. Notably, although this and other studies (Hoff et al. 2026; Bringloe et al. 2024) identified numerous polymorphic candidate CIs, only some contain *F*_ST_ outliers, suggesting different forms of these CIs are not associated with location‐specific adaptation. An exception to this pattern is shown on chromosome 3 where there are two small (~3 MB and ~1.5 MB) putative CIs, both of which are associated with areas with high *F*_ST_. Investigations into local PCAs suggest samples from Chukchi Sea are driving this difference, where most Chukchi Sea samples (~64% and ~89% for each CI) were fixed for the alternative (rare) homokaryotype. Although it should be noted that Hoff et al. (2024) did find evidence of *F*_ST_ outliers within candidate CIs on chromosomes 9 and 16, both of which are variable in the populations sampled herein.

**TABLE 3:** Comparisons of synteny between Arctic cod (*Boreogadus*), polar cod (*Arctogadus*), Atlantic cod ( *Gadus morhua* ), and Atlantic haddock ( *Melanogrammus aeglefinus* ) taken from table S4 in Hoff et al. (2026). Note comparisons of synteny to Atlantic cod were to samples from the northeastern Atlantic population (NEAC).

Arctic cod chromosome (*n* = 18)	Polar cod (*n* = 15)	Atlantic cod (*n* = 23)	Atlantic haddock (*n* = 22)
Bs1a	Ag2b	Ne6	Ma5
Bs1b	Ag4a	Ne11	Ma8
Bs2a	Ag3b	Ne16	Ma2a
Bs2b	Ag5a	Ne20	Ma2b and Ma22
Bs3a	Ag12	Ne3	Ma1a
Bs3b	Ag7a	Ne15	Ma1b
Bs4a	Ag2a	Ne8	Ma12
Bs4b	Ag4b	Ne22	Ma18
Bs5a	Ag6b	Ne17	Ma22
Bs5b	Ag8b	Ne13	Ma14
Bs6	Ag9	Ne4	Ma3
Bs7	Ag10	Ne12	Ma4
Bs8	Ag11	Ne7	Ma6
Bs9	Ag6a	Ne14	Ma7
Bs10	Ag14	Ne10	Ma9
Bs11	Ag1a	Ne1	Ma10
Bs12	Ag15	Ne2	Ma13
Bs13	Ag5b	Ne9	Ma11
Bs14	Ag13	Ne5	Ma16
Bs15	Ag1b	Ne18	Ma17
Bs16	Ag7b	Ne21	Ma19
Bs17	Ag8a	Ne23	Ma15
Bs18	Ag3a	Ne19	Ma20

Past research in other gadids have found strong associations between CIs and adaptive divergence (Kirubakaran et al. 2016; Berg et al. 2017). However, our study suggests that in Arctic cod chromosomal fusions play a larger role in facilitating divergence than CIs at least in the collections sampled herein. It is possible that the CIs identified in Arctic cod may be important for maintaining phenotypic variation within geographic locations (such as has been found in Atlantic cod, e.g., Clucas et al. 2019; Matschiner et al. 2022), but we were unable to investigate relationships between CI karyotypes and phenotypes without associated phenotypic data.

Patterns of genetic differentiation and diversity at fused chromosomes reflect a heterogenous landscape of variation that suggests divergence is more prevalent in fused chromosomes, where alleles have greater potential to accumulate and be selected together. Interestingly, genetic differentiation dropped substantially in fused regions (especially chromosomes 2 and 3). These drops in genetic differentiation did not correspond with consistently high or low LD or nucleotide diversity—although areas of high LD on chromosomes 1 and 2 appear to be centered just outside of the centromere and proximate fusion point (Figure 6). Liu et al. (2021) also observed reduced genetic differentiation at fusion points in stickleback, indicating that this pattern may be more common in fused chromosomes than previously thought. Genetic differentiation was low at the ends of fused chromosomes, which often corresponded with reduced LD. This pattern was also observed by Liu et al. (2021) and likely reflects increased recombination in distal regions of fused chromosomes while the remainder of the chromosome has reduced recombination due to interference near chiasma formation (Vara et al. 2021; Näsvall et al. 2023). A study in *Heliconius* also found broader regions of linkage in recently fused chromosomes compared to unfused, suggesting this might be a common phenomenon for chromosomal fusions (Cicconardi et al. 2021). Nucleotide diversity showed fine‐scale variation across the fused chromosomes with large differences in diversity between Pond Inlet and Chukchi Sea occurring in specific genomic regions. For example, the peak of genetic differentiation near 20 Mb on chromosome 2 contained regions with both negative and positive Δ*Pi* values. Our Δ*Pi* results differ from Liu et al. (2021), where they found largely unidirectional changes in Δ*Pi* on fused chromosomes in a population of stickleback that colonized freshwater when compared to its marine progenitor population. This is supported by the increase in absolute genetic divergence in the majority of these peaks, suggesting that these populations are resistant to introgression and have been separated for enough time to accumulate mutations. These diversity metrics suggest that selective sweeps likely occurred in both the Western and Eastern Canadian cod populations as they have adapted to their respective environments.

Chromosomal fusions are common in gadids and have likely played an important role in speciation and adaptive divergence. The number of chromosomes found across gadid species is variable, with *n* ranging from 13 to 24 (Table 3; Hoff et al. 2026). Notably, both Arctic gadids (i.e., *Arctogadus* and *Boreogadus*) display reduced chromosome numbers compared to species found in more temperate latitudes, indicating that chromosome fusions may have facilitated adaptation to Arctic ecosystems (Hoff et al. 2026). Fusion events were similarly found to be important in the radiation of Notothenioid fishes in Antarctic waters (Auvinet et al. 2020; Amores et al. 2017). Interestingly, Hoff et al. (2026) also identified anti‐freeze glycoproteins on chromosomes 1 and 2, which can enable fishes to withstand freezing temperatures (DeVries 1982). Taken together, these results suggest that chromosomal fusions may be a critical genomic mechanism for adaptations to cold water conditions. However, fusion events did not involve homologous chromosome arms in *Boreogadus* and *Arctogadus*, indicating that fusions occurred independently as these two species colonized Arctic habitats (comparison of syntenic arms in *Arctogadus* and *Boreogadus* are found in Table 3) and might be important in maintaining species boundaries in areas of sympatry. We hypothesize that the chromosomal fusions likely facilitated both speciation and adaptive divergence in Arctic cod and may help explain why populations of Arctic cod are adapted to divergent environments such as those found in the Chukchi Sea and Baffin Bay.

More broadly, our study implicates the importance of chromosomal fusions for promoting local adaptation with gene flow, especially under extreme environmental conditions such as those prevailing in the Arctic. Despite theoretical evidence for the adaptive importance of chromosomal fusions (Guerrero and Kirkpatrick 2014), far fewer studies have found an association between chromosomal fusions and adaptive divergence in wild populations (but see Wellband et al. 2019; Liu et al. 2021; Näsvall et al. 2023). We postulate that this may be due to a lack of genomic resources and comparative genomic studies for non‐model organisms. Uncovering the non‐neutral significance of fusions in both our study and Liu et al. (2021) was facilitated by the newly constructed reference genome and comparative genomic analyses (in our case conducted by Hoff et al. 2026) where sequenced genomes from multiple species were analyzed to understand the history of fusions. Additionally, pangenome studies may also help shed light on the importance of chromosomal fusions within species (e.g., Wellband et al. 2019). We anticipate that the importance of chromosomal fusions will be increasingly revealed with the proliferation of sequenced genomes and comparative genomic studies.

### 4.3 Implications for Arctic Cod Populations in a Changing Climate

Arctic cod are facing changes to their habitats such as increased temperatures, lower sea ice coverage, and altered circumpolar currents (Rantanen et al. 2022). Additionally, northward migrations of competitor species are expected to continue, and these species may outcompete Arctic cod for resources (Wildes et al. 2022). These changes will likely create challenges for a cold‐water specialized species (e.g., Drost et al. 2016; Laurel et al. 2018), especially since Arctic cod are reliant on cold temperatures and sea ice for recruitment. However, more southern populations of Arctic cod may have greater resilience to sub‐Arctic conditions. For example, populations in the southern portion of the Canadian Arctic persist even though they are exposed to large interannual variation in both temperature and the amount of sea ice (Shan et al. 2024). Our study demonstrates that populations in this region have distinct genetic variation localized in fused chromosomes that could be vital for the persistence of the species in the face of numerous stressors. The development of a high‐throughput SNP panel containing a subset of the non‐neutral alleles from these fused chromosomes would be a useful tool for monitoring Arctic cod populations and to track whether alleles associated with lower latitude populations move northwards in response to climate change. This type of movement could lead to increased resilience to changing environments but may also have unanticipated consequences if individuals from southern populations begin interbreeding with individuals from northern populations. Arctic cod are keystone species in the Arctic Ocean food web, and genetic tools will be vital for informing the conservation of this species in the face of drastic environmental challenges.

## Author Contributions

**Matthew C. Hale:** conceptualization (equal). **Natasha Howe:** conceptualization (equal). **Tracey Loewen:** conceptualization (equal). **Kevin Hedges:** conceptualization (equal). **Maxime Geoffroy:** conceptualization (equal). **Jennifer L. Herbig:** conceptualization (equal). **Wes Larson:** conceptualization (equal).

## Funding

Funding for the acquisition of whole genome sequence data was provided by internal funding at Texas Christian University, Fisheries and Oceans Canada, and NOAA Research.

## Conflicts of Interest

The authors declare no conflicts of interest.

## Supporting information


**Table S1:** Sequencing metrics for all samples included in the study, including the name of the associated sequence, the sampling location, sequencing depth, the number of mapped reads, and the percent of reads that mapped, as well as if the depth and read statistics are for downsampled sequences (e.g., Iceland sequences).
**Figure S1:** Whole genome PCA of individuals from all sampling locations from SNPs across the genome.
**Figure S2:** Chromosome‐specific PCAs of individuals from all sampling locations. Most PC axes in unfused chromosomes are influenced by CIs, visualized by the clustering patterns (further investigated in Appendix C).
**Figure S3:** PCAs of the sex‐associated region identified in Hoff (chr5, 26–27.5 Mb). The top left panel shows all individuals colored by collection whereas the bottom left panel shows all individuals but is colored by heterozygosity. The vertical line is the predicted break between males and females. The remaining panels are the same PCA separated by collection, showing that all collections sampled both sexes. Hoff et al. (2024) identified two male haplotypes, which is likely what is occurring in the negative PC1 space separating out on PC2, especially considering that males are expected to have greater heterozygosity, seen here.
**Figure S4:** Manhattan plots of whole genome non‐neutral loci identified in PCAngsd using the FastPCA selection scan, representing PC1 and PC2. Outliers were identified for each PC‐axis with a False Discovery Rate (α = 0.001), The majority of outliers were comprised of SNPs on fused chromosomes (PC1 = 99.4% on fused, PC2 = 55.9% on fused).
**Figure S5:** Heatmap of corresponding weighted *F*_ST_ values from each population comparison in the unfused chromosomes (upper triangle) and fused chromosomes (lower triangle). Labrador was not included due to low sample size.
**Figure S6:** PCA of non‐neutral loci found in unfused chromosomes (chrs 6–18), which shows a similar trend as that of the non‐neutral loci found in fused chromosomes.
**Figure S7:** Nucleotide diversity calculated in 20 kb windows with 10 kb steps separately for Pond Inlet and Chukchi Sea collections. Black points are reflective of estimated centromere locations and gray points are predicted fusion points.
**Appendix A:** Genome‐wide genetic differentiation (*F*_ST_). Manhattan plots of per‐SNP whole genome genetic differentiation (*F*_ST_) of comparison between all collections sampled in this study with a single collection (denoted in the top left of the plot). We did not calculate *F*_ST_ for Labrador Sea due to a small sample size (*n* = 7), whereas all other collections with greater than ten individuals were included. Most regions of high differentiation were found on the first five fused chromosomes, but some additional peaks of differentiation were found on the unfused chromosomes. Horizontal lines represent the Bonferroni‐corrected *p*‐value (α = 0.05) from the *Z*‐score distribution of *F*_ST_ separately for the fused (solid) and unfused (dashed) chromosomes.
**Appendix B:** Admixture. Admixture analyses for the multiple datasets presented in this study tested for two to six ancestral groups. Each column is an individual separated by collection, and each individual's ancestral proportion is represented by the corresponding *K* value. The maximum likelihood (ML) of the number of ancestral populations for *K* = 2 to *K* = 6 is shown in bold.
**Appendix C:** MDS outlier regions—putative inversion testing. Local PCAs for identified MDS outlier regions across *Boreogadus saida* populations. Two individuals with missing data were removed. PCAs of outlier regions were visualized on PC1 vs. PC2 by population (top left) and by relative heterozygosity (bottom left). To assign putative inversion karyotypes, individuals were assigned groups based on their location on PC1, which was split into three equally sized ranges, and individuals in the middle were assigned as heterozygous (0/1) and individuals in the first and third regions were assigned as homozygous (0/0 and 1/1; top right). In many cases, the PCA did not cluster into three groups, so this assignment was not appropriate, but those regions were no longer considered inversions based on our criteria. In two cases (the latter two chr 1 regions), the assignments were slightly altered to reflect the clustering patterns. These groupings were subsequently used for the heterozygosity boxplots (bottom right). Heterozygosity was compared between the two homozygous groups with the heterozygous group using a Wilcoxon rank‐sum test, where heterozygosity was expected to be greater in the middle cluster. *p*‐values (alpha = 0.05) were adjusted via a Benjamini‐Hochberg correction (ns = not significant, *p‐value < 0.05, **p‐value < 0.01, and ***p‐value < 0.001). Some significance results are missing or crossed out as a result of small sample size.
**Appendix D:** Chromosome‐level linkage disequilibrium. Linkage disequilibrium heatmaps are shown for each chromosome in number order for *r* ^2^ values greater than 0.35. Greater values (lighter blue) represent greater linkage, and regions with *r* ^2^ less than 0.35 are not plotted. All individuals were included in these calculations and were not split by geographic region due to small sample sizes. MDS outlier regions are delineated on the diagonal and colored according to whether they were considered a putative CI or not (red = putative CI, pink = unclear clustering, not a putative CI, gray = not a putative CI, teal = sex region).

## Data Availability

Fastq sequence files used in all analyses are available in the SRA database within BioProject accession PRJNA1484137. All computational code used in the analyses of the genomic data is available on Zenodo at https://zenodo.org/records/22284965.

## References

[ece374555-bib-0001] Amores, A. , C. A. Wilson , C. A. H. Allard , H. W. Detrich , and J. H. Postlethwait . 2017. “Cold Fusion: Massive Karyotype Evolution in the Antarctic Bullhead Notothen *Notothenia coriiceps* .” G3: Genes, Genomes, Genetics 7: 2195–2207. 10.1534/g3.117.040063 PMC5498148 28576775

[ece374555-bib-0002] Auvinet, J. , P. Graça , A. Dettai , et al. 2020. “Multiple Independent Chromosomal Fusions Accompanied the Radiation of the Antarctic Teleost Genus *Trematomus* (Notothenioidei: Nototheniidae).” BMC Evolutionary Biology 20, no. 1: 39. 10.1186/s12862-020-1600-3 PMC7082932 32192426

[ece374555-bib-0072] Berg, P. R. , B. Star , C. Pampoulie , et al. 2017. “Trans‐Oceanic Genomic Divergence of Atlantic Cod Ecotypes is Associated With Large Inversions.” Heredity 119, no. 6: 418–428. 10.1038/hdy.2017.54. PMC5677996 28930288

[ece374555-bib-0005] Berner, D. , and M. Roesti . 2017. “Genomics of Adaptive Divergence With Chromosome‐Scale Heterogeneity in Crossover Rate.” Molecular Ecology 26: 6351–6369. 10.1111/mec.14373 28994152

[ece374555-bib-0006] Bolger, A. M. , M. Lohse , and B. Usadel . 2014. “Trimmomatic: A Flexible Trimmer for Illumina Sequence Data.” Bioinformatics 30: 2114–2120. 10.1093/bioinformatics/btu170 PMC4103590 24695404

[ece374555-bib-0007] Bouchard, C. , A. Charbogne , G. Baumgartner , and S. M. Maes . 2020. “West Greenland Ichthyoplankton and How Melting Glaciers Could Allow Arctic Cod Larvae to Survive Extreme Summer Temperatures.” Arctic Science 7: 217–239.

[ece374555-bib-0009] Bouchard, C. , S. Mollard , K. Suzuki , D. Robert , and L. Fortier . 2016. “Contrasting the Early Life Histories of Sympatric Arctic Gadids *Boreogadus saida* and *Arctogadus glacialis* in the Canadian Beaufort Sea.” Polar Biology 39, no. 6: 1005–1022.

[ece374555-bib-0010] Bringloe, T. T. , A. Bourret , D. Cote , et al. 2024. “Genomic Architecture and Population Structure of *Boreogadus saida* From Canadian Waters.” Scientific Reports 13: 19331. 10.1038/s41598-024-69782-w PMC11336163 39164428

[ece374555-bib-0011] Chang, C. C. , C. C. Chow , L. C. A. M. Tellier , S. Vattikuti , S. M. Purcell , and J. J. Lee . 2015. “Second‐Generation PLINK: Rising to the Challenge of Larger and Richer Databases.” GigaScience 4, no. 1: 8. 10.1186/s13742-015-0047-8. PMC4342193 25722852

[ece374555-bib-0012] Cicconardi, F. , J. J. Lewis , S. H. Martin , et al. 2021. “Chromosome Fusion Affects Genetic Diversity and Evolutionary Turnover of Functional Loci, but Consistently Depends on Chromosome Size.” Molecular Biology and Evolution 38, no. 10: 4449–4462. 10.1093/molbev/msab185 PMC8476138 34146107

[ece374555-bib-0065] Clucas, G. V. , R. N. Lou , N. O. Therkildsen , and A. I. Kovach . 2019. “Novel Signals of Adaptive Genetic Variation in Northwestern Atlantic Cod Revealed by Whole‐Genome Sequencing.” Evolutionary Applications 12, no. 10: 1971–1987. 10.1111/eva.12861. PMC6824067 31700539

[ece374555-bib-0014] Constantin, A. , and R. S. Johnson . 2024. “The Dynamics of the Transpolar Drift Current.” Geophysical and Astrophysical Fluid Dynamics 118: 165–182.

[ece374555-bib-0015] Cooper, N. 2022. “NCmisc: Miscellaneous Functions for Creating Adaptive Functions and Scripts.” R Package Version 1.2.0.

[ece374555-bib-0016] David, C. , B. Lange , T. Krumpen , F. Schaafsma , J. A. van Franeker , and H. Flores . 2016. “Under‐Ice Distribution of Polar Cod *Boreogadus saida* in the Central Arctic Ocean and Their Association With Sea‐Ice Habitat Properties.” Polar Biology 39: 981–994.

[ece374555-bib-0017] DeVries, A. L. 1982. “Biological Antifreeze Agents in Coldwater Fishes.” Comparative Biochemistry and Physiology Part A: Physiology 73, no. 4: 627–640.

[ece374555-bib-0018] Drost, H. E. , M. Lo , E. C. Carmack , and A. P. Farrell . 2016. “Acclimation Potential of Arctic Cod ( *Boreogadus saida* ) From the Rapidly Warming Arctic Ocean.” Journal of Experimental Biology 219: 3114–3125. 10.1242/jeb.140194 27471275

[ece374555-bib-0019] Fumagalli, M. , F. G. Vieira , T. Linderoth , and R. Nielsen . 2014. “ngsTools: Methods for Population Genetics Analyses From Next‐Generation Sequencing Data.” Bioinformatics 30, no. 10: 1486–1487. 10.1093/bioinformatics/btu041. PMC4016704 24458950

[ece374555-bib-0020] Gautam, A. 2022. “Phenol‐Chloroform DNA Isolation Method.” In DNA and RNA Isolation Techniques for Non‐Experts, 33–39. Springer.

[ece374555-bib-0021] Geoffroy, M. , C. Bouchard , H. Flores , et al. 2023. “The Circumpolar Impacts of Climate Change and Anthropogenic Stressors on Arctic Cod ( *Boreogadus saida* ) and Its Ecosystem.” Elementa 11: 97. 10.1525/elementa.2022.00097.

[ece374555-bib-0023] Guerrero, R. F. , and M. Kirkpatrick . 2014. “Local Adaptation and the Evolution of Chromosome Fusions.” Evolution 68: 2747–2756. 10.1111/evo.12481 24964074

[ece374555-bib-0024] Hoff, S. N. K. , M. Maurstad , O. K. Tørresen , et al. 2026. “Rapid Genome Modifications Including Chromosomal Fusions and Large‐Scale Inversions Are Key Features in Arctic Codfish Species.” Genome Biology 27: 6. 10.1186/s13059-026-03975-6. PMC13011446 41699629

[ece374555-bib-0025] Hoff, S. N. K. , M. F. Maurstad , A. Le Moan , et al. 2024. “Population Divergence Manifested by Genomic Rearrangements in a Keystone Arctic Species With High Gene Flow.” bioRxiv: The Preprint Server for Biology. 10.1101/2024.06.28.597535.

[ece374555-bib-0026] Hohenlohe, P. A. , W. C. Funk , and O. P. Rajora . 2020. “Population Genomics for Wildlife Conservation and Management.” Molecular Ecology 30: 62–82. 10.1111/mec.15720 PMC7894518 33145846

[ece374555-bib-0027] Huang, K. , R. L. Andrew , G. L. Owens , K. L. Ostevik , and L. H. Rieseberg . 2020. “Multiple Chromosomal Inversion Contribute to Adaptive Divergence of a Dune Sunflower Ecotype.” Molecular Ecology 29: 2535–2549. 10.1111/mec.15428 32246540

[ece374555-bib-0028] Kirubakaran, T. G. , H. Grove , M. P. Ken , S. R. Sandve , and M. Baranski . 2016. “Two Adjacent Inversions Maintain Genomic Differentiation Between Migratory and Stationary Ecotypes of Atlantic Cod.” Molecular Ecology 25: 2130–2143. 10.1111/mec.13592 26923504

[ece374555-bib-0029] Korneliussen, T. S. , A. Albrechtsen , and R. Nielsen . 2014. “ANGSD: Analysis of Next Generation Sequencing Data.” BMC Bioinformatics 15, no. 1: 356. 10.1186/s12859-014-0356-4 PMC4248462 25420514

[ece374555-bib-0030] Laurel, B. J. , L. A. Copeman , M. Spencer , and P. Iseri . 2018. “Comparative Effects of Temperature on Rates of Development and Survival of Eggs and Yolk‐Sac Larvae of Arctic Cod ( *Boreogadus saida* ) and Walleye Pollock ( *Gadus chalcogrammus* ).” ICES Journal of Marine Science 75: 2403–2412. 10.1093/icesjms/fsy042.

[ece374555-bib-0031] Li, H. 2011. “A Statistical Framework for SNP Calling, Mutation Discovery, Association Mapping and Population Genetical Parameter Estimation From Sequencing Data.” Bioinformatics 27, no. 21: 2987–2993. 10.1093/bioinformatics/btr509 PMC3198575 21903627

[ece374555-bib-0032] Li, H. , and R. Durbin . 2009. “Fast and Accurate Short Read Alignment With Burrows‐Wheeler Transform.” Bioinformatics 25: 1754–1760. 10.1093/bioinformatics/btp324 PMC2705234 19451168

[ece374555-bib-0068] Li, H. , and P. Ralph . 2018. “Local PCA Shows How the Effect of Population Structure Differs Along the Genome.” Genetics 211, no. 1: 289–304. 10.1534/genetics.118.301747. PMC6325702 30459280

[ece374555-bib-0033] Liu, Z. , M. Roesti , D. Marques , M. Hiltbrunner , V. Saladin , and C. L. Peichel . 2021. “Chromosomal Fusions Facilitate Adaptation to Divergent Environments in Threespine Stickleback.” Molecular Biology and Evolution 39: msab358. 10.1093/molbev/msab358. PMC8826639 34908155

[ece374555-bib-0034] Lou, R. N. , A. Jacobs , A. P. Wilder , and N. O. Therkildsen . 2021. “A Beginner's Guide to Low‐Coverage Whole Genome Sequencing for Population Genomics.” Molecular Ecology 30, no. 23: 5966–5993. 10.1111/mec.16077. 34250668

[ece374555-bib-0035] Lou, R. N. , and N. O. Therkildsen . 2022. “Batch Effects in Population Genomic Studies With Low‐Coverage Whole Genome Sequencing Data: Causes, Detection and Mitigation.” Molecular Ecology Resources 22, no. 5: 1678–1692. 10.1111/1755-0998.13559. 34825778

[ece374555-bib-0036] Mackintosh, A. , R. Vila , S. H. Martin , D. Setter , and K. Lohse . 2023. “Do Chromosome Rearrangements Fix by Genetic Drift or Natural Selection? Insights From *Brenthis* Butterflies.” Molecular Ecology 33: e17146. 10.1111/mec.17146 PMC11628658 37807966

[ece374555-bib-0037] Maes, S. M. , M. L. Verheye , C. Bouchard , et al. 2025. “Reduced‐Representation Sequencing Detects Trans‐Arctic Connectivity and Local Adaptation in Polar Cod ( *Boreogadus saida* ).” Molecular Ecology 34: e17706. 10.1111/mec.17706 PMC11934089 40040553

[ece374555-bib-0038] Martin, S. H. , J. W. Davey , C. Salazar , and C. D. Jiggins . 2019. “Recombination Rate Variation Shapes Barriers to Introgression Across Butterfly Genomes.” PLoS Biology 17: e2006288. 10.1371/journal.pbio.2006288. PMC6366726 30730876

[ece374555-bib-0066] Matschiner, M. , J. M. I. Barth , O. K. Tørresen , et al. 2022. “Supergene Origin and Maintenance in Atlantic cod.” Nature Ecology & Evolution 6, no. 4: 469–481. 10.1038/s41559-022-01661-x. PMC8986531 35177802

[ece374555-bib-0039] McVean, G. A. T. , S. R. Myers , S. Hunt , P. Deloukas , D. R. Bentley , and P. Donnelly . 2004. “The Fine‐Scale Structure of Recombination Rate Variation in the Human Genome.” Science 304: 581–584. 10.1126/science.1092500 15105499

[ece374555-bib-0064] Meisner, J. , and A. Albrechtsen . 2018. “Inferring Population Structure and Admixture Proportions in Low‐Depth NGS Data.” Genetics 210, no. 2: 719–731. 10.1534/genetics.118.301336. PMC6216594 30131346

[ece374555-bib-0067] Meisner, J. , A. Albrechtsen , and K. Hanghøj . 2021. “Detecting Selection in low‐Coverage High‐Throughput Sequencing Data Using Principal Component Analysis.” BMC Bioinformatics 22, no. 1: 470. 10.1186/s12859-021-04375-2. PMC8480091 34587903

[ece374555-bib-0040] Mulligan, R. P. , and W. Perrie . 2019. “Circulation and Structure of the Mackenzie River Plume in the Coastal Arctic Ocean.” Continental Shelf Research 177: 59–68.

[ece374555-bib-0041] Münchow, A. , K. K. Falkner , and H. Melling . 2015. “Baffin Island and West Greenland Current Systems in Northern Baffin Bay.” Progress in Oceanography 132: 305–317.

[ece374555-bib-0042] Nachman, M. W. 2002. “Variation in Recombination Rate Across the Genome: Evidence and Implications.” Current Opinion in Genetics & Development 12: 657–663. 10.1016/s0959-437x(02)00358-1 12433578

[ece374555-bib-0043] Näsvall, K. , J. Boman , L. Höök , R. Vila , C. Wiklund , and N. Backström . 2023. “Nascent Evolution of Recombination Rate Differences as a Consequence of Chromosomal Rearrangements.” PLoS Genetics 19: e1010717. 10.1371/journal.pgen.1010717. PMC10434929 37549188

[ece374555-bib-0044] Nosil, P. , D. J. Funk , and D. Ortiz‐Barrientos . 2009. “Divergent Selection and Heterogeneous Genomic Divergence.” Molecular Ecology 18: 375–402. 10.1111/j.1365-294X.2008.03946.x 19143936

[ece374555-bib-0045] Ortiz‐Barrientos, D. , J. Engelstadter , and L. H. Rieseberg . 2016. “Recombination Rate Evolution and the Origin of Species.” Trends in Ecology & Evolution 31: P226–P236. 10.1016/j.tree.2015.12.016 26831635

[ece374555-bib-0048] Petty, A. A. , J. K. Hutchings , J. A. Richter‐Menge , and M. A. Tshudi . 2016. “Sea Ice Circulation Around the Beaufort Gyre: The Changing Role of Wind Forcing and the Sea Ice State.” Journal of Geophysical Research: Oceans 121: 3278–3296.

[ece374555-bib-0049] Purcell, S. , B. Neale , K. Todd‐Brown , et al. 2007. “PLINK: A Tool‐Set for Whole‐Genome and Population‐Based Linkage Analyses.” American J of Human Genetics 81, no. 3: 559–575. 10.1086/519795 PMC1950838 17701901

[ece374555-bib-0050] R Core Team . 2023. R: A Language and Environment for Statistical Computing. R Foundation for Statistical Computing.

[ece374555-bib-0051] Rantanen, M. , A. Y. Karpechko , A. Lipponen , et al. 2022. “The Arctic Has Warmed Nearly Four Times Faster Than the Globe Since 1970.” Communications Earth & Environment 3: 168.

[ece374555-bib-0052] Renaut, S. , C. J. Grassa , S. Yeaman , et al. 2013. “Genomic Islands of Divergence Are Not Affected by Geography of Speciation.” Nature Communications 4: 1827. 10.1038/ncomms2833 23652015

[ece374555-bib-0053] Samuk, K. , G. L. Owense , K. E. Delmore , S. E. Miller , D. J. Rennison , and D. Schluter . 2017. “Gene Flow and Selection Interact to Promote Adaptive Divergence in Regions of Low Recombination.” Molecular Ecology 26: 4378–4390. 10.1111/mec.14226 28667780

[ece374555-bib-0054] Schaal, S. M. , B. C. Haller , and K. E. Lotterhos . 2022. “Inversion Invasions: When the Genetic Basis of Local Adaptation Is Concentrated Within Inversions in the Face of Gene Flow.” Philosophical Transactions of the Royal Society, B: Biological Sciences 377: 20210200. 10.1098/rstb.2021.0200. PMC9189506 35694752

[ece374555-bib-0055] Shan, X. , M. A. Spall , C. Pennelly , and P. G. Myers . 2024. “Seasonal Variability in Baffin Bay.” Journal of Geophysical Research: Oceans 129: e2024JC021038. 10.1029/2024JC021038.

[ece374555-bib-0069] Skotte, L. , T. S. Korneliussen , and A. Albrechtsen . 2013. “Estimating Individual Admixture Proportions From Next Generation Sequencing Data.” Genetics 195, no. 3: 693–702. 10.1534/genetics.113.154138. PMC3813857 24026093

[ece374555-bib-0056] Stapley, J. , J. Reger , P. G. D. Feulner , et al. 2010. “Adaptation Genomics: The Next Generation.” Trends in Ecology & Evolution 12: P705–P712. 10.1016/j.tree.2010.09.002 20952088

[ece374555-bib-0057] Straneo, F. , and F. Saucier . 2008. “The Outflow From Hudson Strait and Its Contribution to the Labrador Current.” Deep Sea Research Part I: Oceanographic Research Papers 55: 926–946.

[ece374555-bib-0058] Tigano, A. , and V. L. Friesen . 2016. “Genomics of Local Adaptation With Gene Flow.” Molecular Ecology 25: 2144–2164. 10.1111/mec.13606 26946320

[ece374555-bib-0059] Vara, C. , A. Paytuví‐Gallart , Y. Cuartero , et al. 2021. “The Impact of Chromosomal Fusions on 3D Genome Folding and Recombination in the Germ Line.” Nature Communications 12: 2981. 10.1038/s41467-021-23270-1. PMC8137915 34016985

[ece374555-bib-0060] Welch, H. , M. Bergmann , T. Siferd , et al. 1992. “Energy Flow Through the Marine Ecosystem of the Lancaster Sound Region, Arctic Canada.” Arctic 45: 343–357.

[ece374555-bib-0061] Wellband, K. , C. Mérot , T. Linnansaari , J. A. K. Elliott , R. A. Curry , and L. Bernatchez . 2019. “Chromosomal Fusion and Life‐History Associated Genomic Variation Contribute to Within‐River Local Adaptation of Atlantic Salmon.” Molecular Ecology 28: 1439–1459. 10.1111/mec.14965 30506831

[ece374555-bib-0062] Wellenreuther, M. , and L. Bernatchez . 2018. “Eco‐Evolutionary Genomics of Chromosomal Inversions.” Trends in Ecology & Evolution 33: 427–440. 10.1016/j.tree.2018.04.002 29731154

[ece374555-bib-0070] Wickham, H. 2016. ggplot2: Elegant Graphics for Data Analysis. Springer‐Verlag New York.

[ece374555-bib-0063] Wildes, S. , J. Whittle , H. Nguyen , et al. 2022. “Walleye Pollock Breach the Bering Straight: A Change of the Cods in the Arctic.” Deep Sea Research Part II: Topical Studies in Oceanography 204: 105165. 10.1016/j.dsr2.2022.105165.

[ece374555-bib-0071] Yeaman, S. 2013. “Genomic Rearrangements and the Evolution of Clusters of Locally Adaptive Loci.” Proceedings of the National Academy of Sciences of the United States of America 110, no. 19: E1743–E1751. 10.1073/pnas.1219381110. PMC3651494 23610436

