# Supplementary material for: The Role of Chromosomal Fusions in Genetic Divergence of a Keystone Arctic Species (Boreogadus saida)
Source: Ecol Evol. 2026 Oct 28;16(11):e74555. doi: 10.1002/ece3.74555 (PMC13717224; doi:10.1002/ece3.74555)
Supplement: Supplementary file 1 [file ECE3-16-e74555-s001.docx]

Supplemental Tables

The role of chromosomal fusions in genetic divergence in an Arctic keystone species (*Boreogadus saida*)

Table S1: Sequencing metrics for all samples included in the study, including the name of the associated sequence, the sampling location, sequencing depth, the number of mapped reads, and the percent of reads that mapped, as well as if the depth and read statistics are for downsampled sequences (e.g., Iceland sequences).

| **SampleID** | **Collection Location** | **Depth** | **Mapped Reads** | **Percent Mapped** | **Downsampled** |
| --- | --- | --- | --- | --- | --- |
| AC_01 | Pond Inlet | 13.0851 | 55467941 | 93.07 | N |
| AC_02 | Pond Inlet | 7.75952 | 32019055 | 91.47 | N |
| AC_03 | Pond Inlet | 10.1344 | 42246705 | 91.78 | N |
| AC_04 | Pond Inlet | 8.10067 | 33397314 | 91.87 | N |
| AC_05 | Pond Inlet | 9.88571 | 40821416 | 91.5 | N |
| AC_06 | Pond Inlet | 9.78638 | 40671475 | 91.84 | N |
| AC_07 | Pond Inlet | 9.07107 | 38274642 | 92.16 | N |
| AC_09 | Pond Inlet | 9.46026 | 39558721 | 90.11 | N |
| AC_1008 | Chukchi Sea | 7.51124 | 33392520 | 92.58 | N |
| AC_100 | Frobisher Bay | 11.8456 | 50267701 | 93.93 | N |
| AC_1017 | Chukchi Sea | 7.52148 | 32505161 | 92.09 | N |
| AC_1025 | Chukchi Sea | 7.42325 | 31692831 | 91.39 | N |
| AC_1026 | Chukchi Sea | 8.23253 | 35350618 | 92.06 | N |
| AC_1033 | Chukchi Sea | 7.02072 | 30210835 | 90.59 | N |
| AC_1034 | Chukchi Sea | 8.95774 | 39012254 | 90.53 | N |
| AC_1035 | Chukchi Sea | 7.39214 | 31399278 | 91.96 | N |
| AC_1037 | Chukchi Sea | 9.4957 | 40559044 | 92.03 | N |
| AC_1038 | Chukchi Sea | 9.40763 | 39370998 | 91.18 | N |
| AC_1039 | Chukchi Sea | 8.05611 | 33744201 | 91.41 | N |
| AC_103 | Pond Inlet | 12.4324 | 52180191 | 93.66 | N |
| AC_1040 | Chukchi Sea | 9.29612 | 40091055 | 92.25 | N |
| AC_1041 | Chukchi Sea | 8.59144 | 37051748 | 91.11 | N |
| AC_1043 | Chukchi Sea | 7.7286 | 32808199 | 90.28 | N |
| AC_1044 | Chukchi Sea | 8.72694 | 36949022 | 91.21 | N |
| AC_1047 | Chukchi Sea | 7.85875 | 32878233 | 89.76 | N |
| AC_1048 | Chukchi Sea | 6.90203 | 29789323 | 90.92 | N |
| AC_1049 | Chukchi Sea | 7.72737 | 33189384 | 90.78 | N |
| AC_104 | Pond Inlet | 11.6132 | 48715155 | 93.39 | N |
| AC_105 | Pond Inlet | 10.2346 | 42755248 | 93.59 | N |
| AC_1061 | Chukchi Sea | 7.95051 | 33732537 | 91.54 | N |

| AC_1062 | Chukchi Sea | 7.93034 | 33874946 | 90.8 | N |
| --- | --- | --- | --- | --- | --- |
| AC_1068 | Chukchi Sea | 7.69408 | 31659049 | 91.01 | N |
| AC_106 | Pond Inlet | 10.3565 | 43418395 | 93.89 | N |
| AC_1074 | Chukchi Sea | 7.75406 | 31886677 | 89.42 | N |
| AC_107 | Frobisher Bay | 8.17817 | 34232541 | 92.4 | N |
| AC_1082 | Chukchi Sea | 8.29018 | 35287335 | 90.85 | N |
| AC_1083 | Chukchi Sea | 8.55134 | 36102264 | 91.33 | N |
| AC_1084 | Chukchi Sea | 7.76174 | 32945269 | 92.07 | N |
| AC_1085 | Chukchi Sea | 7.45635 | 32092867 | 92.36 | N |
| AC_1086 | Chukchi Sea | 6.8345 | 29438770 | 90.61 | N |
| AC_1087 | Chukchi Sea | 8.51758 | 36633114 | 91.83 | N |
| AC_1088 | Chukchi Sea | 7.99889 | 32767805 | 91.07 | N |
| AC_1089 | Chukchi Sea | 6.85519 | 28813907 | 91.61 | N |
| AC_1090 | Chukchi Sea | 8.39886 | 34930272 | 89.9 | N |
| AC_1091 | Chukchi Sea | 8.3457 | 34836383 | 88.23 | N |
| AC_1092 | Chukchi Sea | 8.56267 | 35455577 | 91.16 | N |
| AC_1093 | Chukchi Sea | 7.72837 | 32155464 | 90.16 | N |
| AC_1094 | Chukchi Sea | 8.25612 | 34340711 | 88.7 | N |
| AC_1095 | Chukchi Sea | 7.68573 | 32323243 | 90.52 | N |
| AC_1096 | Chukchi Sea | 6.73479 | 28375672 | 90.19 | N |
| AC_10 | Pond Inlet | 8.76702 | 37181973 | 92.79 | N |
| AC_113 | Frobisher Bay | 9.30053 | 39463571 | 92.81 | N |
| AC_114 | Frobisher Bay | 7.25473 | 30498232 | 92.57 | N |
| AC_115 | Frobisher Bay | 8.88491 | 37521299 | 92.92 | N |
| AC_116 | Frobisher Bay | 9.90714 | 41200094 | 91.77 | N |
| AC_117 | Frobisher Bay | 9.61468 | 39813597 | 92.47 | N |
| AC_119 | Frobisher Bay | 7.99054 | 33607913 | 92.66 | N |
| AC_124 | Coronation Gulf | 7.77652 | 33501561 | 92.23 | N |
| AC_126 | Coronation Gulf | 7.59779 | 32416400 | 93.34 | N |
| AC_127 | Coronation Gulf | 7.97479 | 33609095 | 92.06 | N |
| AC_129 | Coronation Gulf | 8.48446 | 35614162 | 89.75 | N |
| AC_12 | Pond Inlet | 9.18514 | 38584868 | 92.37 | N |
| AC_132 | Coronation Gulf | 8.77933 | 36662707 | 91.98 | N |
| AC_136 | Pond Inlet | 8.63467 | 36137128 | 92.33 | N |
| AC_137 | Broughton Island | 11.1153 | 46672178 | 92.35 | N |
| AC_138 | Broughton Island | 8.56944 | 35423601 | 91.78 | N |
| AC_139 | Broughton Island | 10.1787 | 41942950 | 91.88 | N |
| AC_13 | Pond Inlet | 10.0651 | 44219149 | 93.96 | N |
| AC_140 | Broughton Island | 8.15421 | 34226165 | 92.45 | N |
| AC_141 | Broughton Island | 10.2905 | 42975273 | 92.07 | N |
| AC_142 | Broughton Island | 8.98295 | 37728267 | 92.2 | N |
| AC_144 | Broughton Island | 8.24928 | 34556432 | 91.82 | N |
| AC_145 | Broughton Island | 9.0345 | 37997551 | 92.01 | N |

| AC_146 | Broughton Island | 8.75563 | 36477521 | 91.79 | N |
| --- | --- | --- | --- | --- | --- |
| AC_147 | Broughton Island | 8.92419 | 37151171 | 92.09 | N |
| AC_148 | Broughton Island | 6.53215 | 27325212 | 92.28 | N |
| AC_149 | Broughton Island | 8.92263 | 37473012 | 92.26 | N |
| AC_14 | Coronation Gulf | 13.7218 | 58086907 | 92.54 | N |
| AC_152 | Broughton Island | 8.3905 | 36038043 | 91.19 | N |
| AC_15 | Coronation Gulf | 8.68891 | 37075965 | 93.88 | N |
| AC_161 | Broughton Island | 9.52612 | 39829396 | 91.87 | N |
| AC_162 | Broughton Island | 7.75086 | 32462851 | 92.26 | N |
| AC_164 | Pond Inlet | 9.49645 | 39367131 | 92.35 | N |
| AC_165_2 | Pond Inlet | 8.60176 | 36446273 | 92.63 | N |
| AC_165 | Pond Inlet | 11.2546 | 46634854 | 92.32 | N |
| AC_166 | Pond Inlet | 8.95032 | 37232209 | 92.15 | N |
| AC_167_2 | Pond Inlet | 9.02445 | 38632967 | 93.2 | N |
| AC_167 | Pond Inlet | 10.1872 | 42501405 | 92.27 | N |
| AC_168 | Pond Inlet | 9.50398 | 39628060 | 92.56 | N |
| AC_170 | Pond Inlet | 7.89169 | 33563243 | 92.02 | N |
| AC_19 | Coronation Gulf | 9.23496 | 39613432 | 93.83 | N |
| AC_21 | Coronation Gulf | 11.3204 | 46704053 | 90.2 | N |
| AC_34 | Frobisher Bay | 8.19573 | 35078918 | 93.67 | N |
| AC_36 | Frobisher Bay | 10.8413 | 46254056 | 94.04 | N |
| AC_40 | Southampton Island | 8.18617 | 34936201 | 94.27 | N |
| AC_44 | Southampton Island | 8.87418 | 37938885 | 94.06 | N |
| AC_460 | Pond Inlet | 6.54282 | 27956206 | 93.36 | N |
| AC_46 | Southampton Island | 14.2071 | 60373157 | 93.74 | N |
| AC_47 | Southampton Island | 8.97032 | 36857797 | 91.91 | N |
| AC_6509 | Labrador Sea | 9.84389 | 45014895 | 94.17 | N |
| AC_6510 | Labrador Sea | 9.97991 | 44990177 | 93.75 | N |
| AC_6517 | Labrador Sea | 9.73218 | 41310028 | 93.52 | N |
| AC_6520 | Labrador Sea | 11.2736 | 52097685 | 93.78 | N |
| AC_6525 | Labrador Sea | 8.85274 | 40457783 | 94.21 | N |
| AC_6529 | Labrador Sea | 7.93603 | 36594136 | 94.19 | N |
| AC_6544 | Hudson Strait | 7.7334 | 33345455 | 92.64 | N |
| AC_6552 | Hudson Strait | 8.96721 | 37679418 | 93.06 | N |
| AC_6555 | Hudson Strait | 10.7684 | 45293043 | 93.45 | N |
| AC_6557 | Hudson Strait | 8.70057 | 36518286 | 93.25 | N |
| AC_6560 | Hudson Strait | 9.59661 | 40667982 | 93.71 | N |
| AC_6561 | Hudson Strait | 10.1527 | 42963485 | 93.54 | N |
| AC_6568 | Hudson Strait | 11.923 | 50903438 | 93.48 | N |
| AC_6569 | Hudson Strait | 10.6844 | 45246122 | 93.31 | N |
| AC_6570 | Hudson Strait | 11.1406 | 47321059 | 93.44 | N |
| AC_6573 | Hudson Strait | 7.38134 | 31547407 | 93.24 | N |
| AC_6582 | Hudson Strait | 5.61624 | 24362057 | 93.44 | N |

| AC_6585 | Hudson Strait | 9.67154 | 41027002 | 93.45 | N |
| --- | --- | --- | --- | --- | --- |
| AC_6587 | Hudson Strait | 8.2971 | 35284179 | 93.35 | N |
| AC_6588 | SE Baffin | 10.5338 | 45014278 | 93.58 | N |
| AC_6589 | SE Baffin | 8.50849 | 36191372 | 93.24 | N |
| AC_6590 | SE Baffin | 10.3094 | 43521962 | 93.48 | N |
| AC_6591 | SE Baffin | 9.11779 | 38421067 | 93.32 | N |
| AC_6602 | SE Baffin | 10.7291 | 47431635 | 93.33 | N |
| AC_6606 | SE Baffin | 10.1641 | 43263577 | 93.29 | N |
| AC_6607 | SE Baffin | 9.43713 | 40153345 | 93.78 | N |
| AC_6611 | SE Baffin | 10.8167 | 46233537 | 93.65 | N |
| AC_6614 | SE Baffin | 11.1876 | 46867350 | 91.47 | N |
| AC_6628 | SE Baffin | 7.49635 | 31896931 | 93.07 | N |
| AC_6630 | SE Baffin | 9.8323 | 41668313 | 93.6 | N |
| AC_6632 | SE Baffin | 8.50268 | 35833400 | 93.53 | N |
| AC_6637 | Labrador Sea | 9.52528 | 40611800 | 93.74 | N |
| AC_71 | Pond Inlet | 11.2882 | 47788061 | 93.36 | N |
| AC_73 | Coronation Gulf | 11.0649 | 47520308 | 93.89 | N |
| AC_74 | Coronation Gulf | 10.294 | 42565378 | 92.19 | N |
| AC_75 | Coronation Gulf | 8.64437 | 39054193 | 94.12 | N |
| AC_76 | Frobisher Bay | 13.2923 | 56265230 | 93.57 | N |
| AC_77 | Frobisher Bay | 11.0359 | 47619861 | 93.85 | N |
| AC_80 | Frobisher Bay | 11.8175 | 50047512 | 93.24 | N |
| AC_82 | Frobisher Bay | 11.319 | 47771044 | 93.35 | N |
| AC_83 | Frobisher Bay | 11.6141 | 50181473 | 93.61 | N |
| AC_90 | Southampton Island | 11.3191 | 46891524 | 92.29 | N |
| AC_91 | Southampton Island | 13.7583 | 58560782 | 94.1 | N |
| AC_92 | Southampton Island | 13.573 | 55825098 | 92.33 | N |
| AC_93 | Southampton Island | 13.9672 | 58305900 | 92.95 | N |
| AC_94 | Southampton Island | 13.1875 | 55357934 | 93.85 | N |
| AC_95 | Southampton Island | 11.0989 | 46826658 | 94.37 | N |
| AC_97 | Southampton Island | 11.5615 | 48797964 | 94.13 | N |
| AC_98 | Southampton Island | 9.03701 | 38670160 | 94.69 | N |
| AC_99 | Southampton Island | 10.4741 | 44879285 | 94.98 | N |
| PC_06 | Frobisher Bay | 10.0788 | 41960687 | 92.07 | N |
| PC_07 | Frobisher Bay | 8.20651 | 34180270 | 90.85 | N |
| PC_08 | Frobisher Bay | 8.04029 | 33451172 | 91.91 | N |
| Ice_cod10 | Iceland | 8.49119 | 35168082 | 96.13 | Y |
| Ice_cod11 | Iceland | 7.69967 | 32155111 | 95.69 | Y |
| Ice_cod12 | Iceland | 9.30597 | 38646642 | 96.33 | Y |
| Ice_cod13 | Iceland | 8.27734 | 34617311 | 96.27 | Y |
| Ice_cod14 | Iceland | 8.86743 | 36982882 | 96.1 | Y |
| Ice_cod15 | Iceland | 9.38937 | 39178738 | 96.18 | Y |
| Ice_cod16 | Iceland | 9.37775 | 38795475 | 96.23 | Y |

| Ice_cod17 | Iceland | 11.1507 | 46403940 | 96.4 | Y |
| --- | --- | --- | --- | --- | --- |
| Ice_cod18 | Iceland | 9.34103 | 39013063 | 95.89 | Y |
| Ice_cod1 | Iceland | 8.79031 | 36456930 | 94.76 | Y |
| Ice_cod2 | Iceland | 13.7821 | 57652206 | 94.23 | Y |
| Ice_cod3 | Iceland | 9.54963 | 39677543 | 96.44 | Y |
| Ice_cod4 | Iceland | 8.92383 | 37186251 | 96.39 | Y |
| Ice_cod5 | Iceland | 9.92714 | 41323862 | 96.44 | Y |
| Ice_cod6 | Iceland | 7.97458 | 33079640 | 96.17 | Y |
| Ice_cod7 | Iceland | 10.2581 | 42816332 | 96.19 | Y |
| Ice_cod8 | Iceland | 8.25044 | 34185857 | 95.97 | Y |
| Ice_cod9 | Iceland | 9.56481 | 40064831 | 93.93 | Y |

Supplemental Figures

###### The role of chromosomal fusions in genetic divergence in an Arctic keystone species (*Boreogadus saida*)

**Figure S1**: Whole genome PCA of individuals from all sampling locations from SNPs across the genome.

**Figure S2**: Chromosome-specific PCAs of individuals from all sampling locations. Most PC axes in unfused chromosomes are influenced by

putative CIs, visualized by the clustering patterns (further investigated in Appendix C).

**Figure S3**: PCAs of the sex-associated region identified in Hoff (chr5, 26-27.5Mb). The top left panel shows all individuals colored by collection whereas the bottom left panel shows all individuals but is colored by heterozygosity. The vertical line is the predicted break between males and females. The remaining panels are the same PCA separated by collection, showing that all collections are represented by both sexes. Hoff et al (2024) identified two male haplotypes, which is likely what is occurring in the negative PC1 space separating out on PC2, especially considering that males are expected to have greater heterozygosity, seen here.

**Figure S4**: Manhattan plots of whole genome adaptive loci identified in PCAngsd using the FastPCA selection scan, representing PC1 and PC2. Outliers were identified for each pc-axis with a False Discovery Rate (α = 0.001), Most outliers were comprised of SNPs on fused chromosomes (PC1 = 99.4% on fused, PC2 = 55.9% on fused).

**Figure S5**: Heatmap of corresponding weighted *F*ST values from each location comparison in the unfused chromosomes (upper triangle) and fused chromosomes (lower triangle). Labrador was not included due to the low sample size.

**Figure S6:** PCA of adaptive loci found in unfused chromosomes (chrs 6-18), which shows a similar trend as that of the adaptive loci found in fused chromosomes.

**Figure S7**: Nucleotide diversity calculated in 20kb windows with 10kb steps separately for Pond Inlet and Chukchi Sea collections. Black points are reflective of estimated centromere locations and gray points are predicted fusion points.

### Supplemental Appendices

###### The role of chromosomal fusions in genetic divergence in an Arctic keystone species (*Boreogadus saida*)

***Appendix A:*** *Genome-wide Genetic Diﬀerentiation (FST)*

Manhattan plots of per-SNP whole genome genetic diﬀerentiation (*F*ST) of comparison between all collections sampled in this study with a single collection (denoted in the top left of the plot). We did not calculate *F*ST for Labrador Sea due to a small sample size (n=7), whereas all other collections with greater than ten individuals were included. Most regions of high diﬀerentiation were found on the ﬁrst ﬁve fused chromosomes, but some additional peaks of diﬀerentiation were found on the unfused chromosomes. Horizontal lines represent the Bonferroni-corrected

𝘱𝘱-value (𝛼𝛼 = 0.05) from the z-score distribution of *F*ST separately for the fused (solid) and unfused (dashed) chromosomes.

***Appendix B:*** *Admixture*

Admixture analyses for the multiple datasets presented in this study tested for two to six ancestral groups. Each column is an individual separated by collection, and each individual’s ancestral proportion is represented by the corresponding K value. The maximum likelihood (ML) of number of ancestral populations for K=2 to K=6 is shown in bold and in the description below:

i) **Whole genome** (ML K=2) – SNPs across the genome
ii) **Fused chromosomes: All Loci** (ML K=4) – All SNPs found on chromosomes 1-5
iii) **Fused chromosomes: Adaptive Loci** (ML K=4) – SNPs found on chromosomes 1-5

that passed the FST ﬁltering cutoﬀ as described in the methods

iv) **Unfused chromosomes: All Loci** (ML K=2) – All SNPs found on chromosomes 6-18
v) **Unfused chromosomes: Neutral Loci** (ML K=2) – SNPs found on chromosomes 6-18 that passed the FST ﬁltering cutoﬀ and were not found in putative inversions, as described in the methods
vi) **Unfused chromosomes: Adaptive Loci** (ML K=3) – SNPs found on chromosomes 6-58 that passed the FST ﬁltering cutoﬀ as described in the methods.

**K2** 1.0

0.8

0.6

0.4

0.2

K31.0

0.8

0.6

0.4

0.2

K41.0

0.8

0.6

0.4

0.2

K51.0

0.8

0.6

0.4

0.2

K61.0

0.8

0.6

0.4

0.2

Chukchi

Coronation

Pond

Broughton

SEBaffin

Frobisher

Labrador

Hudson

Southampton

Iceland

## Whole Genome

Cluster K1 K2

K3

K4 K5 K6

K21.0

0.8

0.6

0.4

0.2

K31.0

0.8

0.6

0.4

0.2

**K4** 1.0

0.8

0.6

0.4

0.2

K51.0

0.8

0.6

0.4

0.2

K61.0

0.8

0.6

0.4

0.2

Chukchi

Coronation

Pond

Broughton

SEBaffin

Frobisher

Labrador

Hudson

Southampton

Iceland

## Fused: All Loci

Cluster K1 K2

K3

K4 K5 K6

K21.0

0.8

0.6

0.4

0.2

K31.0

0.8

0.6

0.4

0.2

**K4** 1.0

0.8

0.6

0.4

0.2

K51.0

0.8

0.6

0.4

0.2

K61.0

0.8

0.6

0.4

0.2

Chukchi

Coronation

Pond

Broughton

SEBaffin

Frobisher

Labrador

Hudson

Southampton

Iceland

## Fused: Adaptive Loci

Cluster K1 K2

K3

K4 K5 K6

**K2** 1.0

0.8

0.6

0.4

0.2

K31.0

0.8

0.6

0.4

0.2

K41.0

0.8

0.6

0.4

0.2

K51.0

0.8

0.6

0.4

0.2

K61.0

0.8

0.6

0.4

0.2

Chukchi

Coronation

Pond

Broughton

SEBaffin

Frobisher

Labrador

Hudson

Southampton

Iceland

## Unfused: All Loci

Cluster K1 K2

K3

K4 K5 K6

**K2** 1.0

0.8

0.6

0.4

0.2

K31.0

0.8

0.6

0.4

0.2

K41.0

0.8

0.6

0.4

0.2

K51.0

0.8

0.6

0.4

0.2

K61.0

0.8

0.6

0.4

0.2

Chukchi

Coronation

Pond

Broughton

SEBaffin

Frobisher

Labrador

Hudson

Southampton

Iceland

## Unfused: Neutral Loci

Cluster K1 K2

K3

K4 K5 K6

K21.0

0.8

0.6

0.4

0.2

**K3** 1.0

0.8

0.6

0.4

0.2

K41.0

0.8

0.6

0.4

0.2

K51.0

0.8

0.6

0.4

0.2

K61.0

0.8

0.6

0.4

0.2

Chukchi

Coronation

Pond

Broughton

SEBaffin

Frobisher

Labrador

Hudson

Southampton

Iceland

## Unfused: Adaptive Loci

Cluster K1 K2

K3

K4 K5 K6

***Appendix C:*** *MDS Outlier Regions – Putative Inversion Testing*

Local PCAs for identiﬁed MDS outlier regions across *Boreogadus saida* populations. Two individuals with missing data were removed. PCAs of outlier regions were visualized on PC1 vs. PC2 by population (top left) and by relative heterozygosity (bottom left). To assign putative inversion karyotypes, individuals were assigned groups based on their location on PC1, which was split into three equally sized ranges, and individuals in the middle were assigned as heterozygous (0/1) and individuals in the ﬁrst and third regions were assigned as homozygous (0/0 and 1/1; top right). In many cases, the PCA did not cluster into three groups, so this assignment wasn’t appropriate, but those regions were no longer considered inversions based on our criteria. In two cases (the latter two chr 1 regions), the assignments were slightly altered to reﬂect the clustering patterns. These groupings were subsequently used for the heterozygosity boxplots (bottom right). Heterozygosity was compared between the two homozygous groups with the heterozygous group using a Wilcoxon rank-sum test, where heterozygosity was expected to be greater in the middle cluster. P-values were adjusted via a Benjamini-Hochberg correction (ns = not signiﬁcant, * p-value < 0.05, **p-value < 0.01, and *** p-value < 0.001). Some signiﬁcance results are missing or crossed out as a result of small sample size.

Unclear groups, potentially two SVs with six groups

Adjusted groupings, potentially only 2 types

ns

Potentially 2 SVs with 6 groups but unclear

MOS Outlier Region - chr 2 : 46.2 - 48.9 Mb

••

'••

•

0.1

Populations	•

- •	• Chukchi	0.1	**4**	• •

�-

- _•_	••• • '
- Coronation
  - •• • • '

•	•	•	_-�_•- ••

•

- Pond

\• •··

•	•	•. ••

0.0

u**N**

••

*�:*^•^

.._•_..,• •	••

- Broughton	0.0
- SEBaffin	u**N**

•••

•

._•_..., • •	••

a..

.,. I •	••

- Frobisher

a..

...,....	.. _•_	••

-0.1

•..•

•

- Labrador

•	•

-0.1	.......,	•	•

••	•	•

Hudson

•

•.. •	•

• •	•

Southampton

- Iceland

• •	•

-0.2

'••

•

•	-0.2	•

0.0	0.1	0.2	0.0	0.1	0.2

PC1	PC1

0.4

0.1	**_4_**

- • • •'

Relative

\•·

- • •	. • . ••

Heterozygosity

->- 0.3

• •·

. . • • • •

1.00

"iii

0.0	�••-•	•.	•	**^0^**

u**N**

--=-•..

._•_,

0.75

**C)**

>,**N**

E;:3 0/0

E;:3

a,

- IQ)

a..

""'

E;:3

.,,... • _•_	• •

0.50

-**^0^** 0.2

0/1

-0.1

......., •	•

1/1

0.25

0.00	0.1

• •	•

..

•

-0.2

***

***

•

•

•

•

- 0.0

0.0	0.1	0.2	0/0	0/1	1/1

PC1	Putative Inversion Type

MOS Outlier Region - chr 3 : 34.2 - 35.9 Mb

0.4

0.2

u**N**

Populations	0.4

- Chukchi
- Coronation
- Pond
- Broughton	0.2
- SEBaffin	u**N**

a..

- Frobisher

a..

0.0

Labrador

- Hudson	0.0
- Southampton
- Iceland

-0.05	0.00	0.05	0.10	-0.05	0.00	0.05	0.10

PC1	PC1

0.4

0.2

Relative Heterozygosity

1.00

0.4

>- 0.3

-

"iii

**0**

**N**	0.75	**^C)^**

>,

u

**N**

#### E;:3

0/0

a..

0.50

-a,**^0^** 0.2

#### E;:3

0/1

0.0

0.25	IQ)

0.00

0.1

0.0

E;:3 1/1

-0.05	0.00	0.05	0.10	0/0	0/1	1/1

• **·f**•

•

**f**

•

• •

**•**

•

•

•

•.,

•

•

*I*

•

•

•

•

**f**

•

.,

••

***J***_•_

- **J.**

•

-\_•_

•

**•**

*I*

**f**

•

•

•

•

**·f**•

•

.,

••

***J***_•_

- **J.**

•

-\_•_

*I*

***

***

•

_+_•

i

I

•

PC1	Putative Inversion Type

MOS Outlier Region - chr 4 : 20.4 - 25.5 Mb

0.2	*• ••*

0.2

Unclear clustering, likely not correctly grouping. 0/1 & 1/1 might all be 0/1

*• ••*

*•• •*,,*•*	,.

Populations

*_••_ _•_*,,,*_•_*	,.

0.1

*• •*

,

.. *•*

*^•^* ·*•*-·

*•	••*

*_•_*�·

- Chukchi
- Coronation

0.1

*•*

- Pond	.

..,

=·..,

*• •*

.*•* ·*•*-·

- *••*...

ua..

**N**

0.0

*•*

- Broughton

*•	•• • •*

- SEBaffin	ua..

0.0

**� -11.**	*•*

*• •*

**f**

**N**

�-- ,-

*• •*

*•• • •*

-0.1

*• •*

*• •*

*•	•	• •*

*•*ll *•*• •

*•	• •*

*•	•*

*•	•*

*••	•	•*

*••	•*

Labrador

- Frobisher
  - Hudson	-0.1
  - Southampton

,

*_•_*'	*•	• •*

- *•	•	•*	,

*••	•*

,,.'•

-0.2

- *•* Iceland	*•*

*•*	-0.2	*•*

0.0	0.1	0.2	0.0	0.1	0.2

PC1	PC1

0.2	*• ••*

*•• •*,,,*•*	..,..

·-·

0.4

0.1

*• •	•	••*

*^•^*. *•* ..,	=· *•*

..,

.-,-

Relative Heterozygosity

->- 0.3

**N** 0.0

u

a..

�-

**� -11.**

*•• • •	•*

*•	•	•*

*•*

1.00

0.75

"iii

**0**

**C)**

>,

##### E;:3

*E;:3*

0/0

*_•_ •*	'•

*•*

*•*

*•	• •*

0.50

-**^0^** 0.2

##### E;:3

0/1

-0.1

**t;**' **-=** *•*

,,.*•*

*••	•*

- *•*	,

*•*

a,

Q)

0.25

1/1

*•	•	•*

*•*

*•*

-0.2

0.00	0.1

0.0

0.0	0.1	0.2	0/0	0/1	1/1

***

ns

I

*•*

PC1	Putative Inversion Type

MOS Outlier Region - chr 5 : 18.3 - 18.9 Mb

•	•

•

0.1

•-•^•^. •

Populations

- Chukchi

u

,_•_..

- Coronation

0.1

-.-,.,.·

•-•^•^.•^•^

•

.._•_,=-•.-•

•

- •	Pond

t. ��-

._•_,-•.-•	•

**N** 0.0

u

a..

•

•••

•

... '"-	•	Broughton SEBaffin

- Frobisher

•

**N** 0.0

a..

--�·

...***c***..***:*** .

_•_ **•**..•.

•

•

... ,...

-0.1

-0.2

0.1

^•^*:,*^•^

•

,•.•,-.

-�·

•

..•.

.

0.0

- "'«>

0.1

PC1

•-•^•^.•^•^

•

0.2

Labrador

- Hudson
- Southampton
- Iceland

-0.1

-0.2

I•'

^•^*:,*^•^

•

0.0

- **-.t,**

0.1

PC1

0.2

0.0

**t**-**�-**

_•_,-•.•_

•

...

,...

**N**

u

a..

-0.1

-0.2

..***c***•***:***

I•

^•^*:,*^•^

•

0.0

- • ...

•

- **-.t,**

0.1

0.2

0.0

0/0

0/1

1/1

E;:3 0/0

E;:3 0/1

|  | Relative  Heterozygosity  1.00 | 0.4  *·*-*w*>- 0.3 |
| --- | --- | --- |
|  | 0.75 | **0**  **C)**  **N** |
|  | 0.50 | -a,**^0^** 0.2 |
|  | 0.25 | IQ) |
|  | 0.00 | 0.1 |

E;:3 1/1

PC1	Putative Inversion Type

***

$

•

***

•

•

This is the sex region, not an inversion

Pink is putative males [0/1], Blue is putative females [1/1]

Sex

Potentially two SVs with six clusters

Potentially two SVs with six clusters

Unclear clustering patterns

MOS Outlier Region - chr 11 : 0.1 - 8.7 Mb

••••

Does not look like an SV pattern, but Chukchi driven.

**. ·**-**s**•

•

0.0	•	•

•

Populations

- • Chukchi

0.0

\ •	•	•

- Coronation
- Pond
- Broughton

u**N**	• SEBaffin	u**N**

• •	•

a_ -0.2

-0.4	•

- •	Frobisher Labrador
  - Hudson
  - Southampton

a_ -0.2	•

-0.4	•

- •	•	Iceland	••

•	•

0.0	0.1	0.2	0.3	0.0	0.1	0.2	0.3

PC1	PC1

0.0

u**N**

\_•_ _•_. _•_

•

- •	Relative Heterozygosity

**. ·**-**s**•

1.00

0.75

0.4

>- 0.3

-

"iii

**0**

**C)**

-

**N**>,

a,

#### E;:3

E;:3

0/0

a_ -0.2

- 0.50

0.25

**0** 0.2

#### E;:3

0/1

1/1

-0.4

• ••

0.00	0.1

- 0.0

•

-!-_•_

I

•

0.0	0.1	0.2	0.3	0/0	0/1	1/1

PC1	Putative Inversion Type

Interestingly, heterozygosity differs across all three

Potentially two SVs - six clusters

Unclear if three or six groups

***Appendix D:*** *Chromosome-Level Linkage Disequilibrium*

Linkage disequilibrium heatmaps are shown for each chromosome in number order for r^2^ values greater than 0.35. Greater values (lighter blue) represent greater linkage, and regions with r^2^ less than 0.35 are not plotted. All individuals were included in these calculations and were not split by geographic region due to small sample sizes.

MDS outlier blocks added to corresponding chromosome-level LD heatmap.

**Chr 1**

**Chr 2**

**Chr 3**

**Chr 4**

**Chr 5**

**Chr 6**

**Chr 7**

**Chr 8**

**Chr 9**

**Chr 10**

**Chr 11**

**Chr 12**

# Chr 13

**Chr 14**

**Chr 15**

**Chr 16**

**Chr 17**

**Chr 18**
